# A Classification Recognition Method of UAVs and Birds Based on a Fusion Measure with the VMD-Driven Model and Time–Frequency Spectrum Segmentation

**DOI:** 10.3390/s26196113

**Published:** 2026-09-26

**Authors:** Siqi Peng, Hanshan Li

**Affiliations:** 1 School of Intelligent Science and Engineering, Xi’an Peihua University, Xi’an 710125, China; 003058@peihua.edu.cn; 2 School of Electronic and Information Engineering, Xi’an Technological University, Xi’an 710021, China

**Keywords:** unmanned aerial vehicle (UAV), micro-Doppler, variational mode decomposition (VMD), short-time Fourier transform (STFT), feature fusion, weighted Gaussian Naive Bayes

## Abstract

**Highlights:**

**What are the main findings?**

- We propose a two-domain micro-Doppler feature extraction framework based on VMD modal features and STFT subband statistical features.
- We develop a weighted Gaussian Naive Bayes classifier using the selected VMD modal energy ratio and STFT subband mean features.

**What are the implications of the main findings?**

- The first major finding is that the VMD and STFT feature domains provide complementary descriptions of UAV and bird micro-motion characteristics.
- The second major finding is that the proposed framework maintains competitive classification performance under the evaluated low-SNR conditions.

**Abstract:**

To improve the classification and recognition ability of radar detection unmanned aerial vehicles (UAV) under low signal-to-noise ratio conditions, this paper propose a two-domain feature extraction method that combines variational mode decomposition (VMD)-based modal features with short-time Fourier transform (STFT)-based subband statistical features. The modal energy ratio and subband mean are selected as representative features from the two domains and are normalized and fused to construct the final feature vector. A weighted Gaussian Naive Bayes classifier is then proposed for UAV–bird discrimination. The feature weights are estimated from the training data, and the final classification decision is obtained using the maximum a posteriori criterion. Experimental results under different low-SNR conditions show that the proposed method maintains competitive classification performance and retains useful discriminative information under strong noise interference. The results indicate that the combination of VMD modal features and STFT subband statistical features provides an effective representation for UAV–bird classification under low-SNR conditions.

## 1. Introduction

With the rapid development and widespread deployment of unmanned aerial vehicles (UAVs), low-altitude airspace has become increasingly complex, creating new challenges for airspace surveillance and security. UAVs have attracted considerable attention because of their flexibility, maneuverability, low operating cost, and diverse application capabilities [1,2]. Recent studies have investigated UAV detection using learning-based and vision-based methods [3,4,5]. RSSI-based approaches have also been explored for anomalous drone detection [6]. In radar-based applications, millimeter-wave ISAR imaging and radar detection probability models have been studied for UAV detection and analysis [7,8]. However, the small radar cross section, low flight speed, and complex motion characteristics of UAVs still make reliable radar detection and identification difficult, particularly under unfavorable signal-to-noise ratio conditions. Therefore, accurate detection and classification of small UAV targets have become important research topics in low-altitude radar surveillance.

In practical low-altitude surveillance scenarios, UAVs and birds are both typical low-altitude, slow-speed, and small-scale (LSS) targets, and their overall Doppler characteristics may partially overlap. As a result, birds can generate false alarms in UAV surveillance systems, making the discrimination between UAVs and birds particularly important. Radar micro-Doppler signatures provide valuable information for this task because rotating UAV blades and flapping bird wings introduce different time-varying frequency modulations into the radar echo. However, as the SNR decreases, these weak micro-motion components are increasingly masked by noise and interference, resulting in attenuation, fragmentation, and even disappearance of discriminative time–frequency structures. Therefore, how to construct informative and complementary features from weak micro-Doppler echoes remains an important problem for UAV–bird classification under low-SNR conditions.

To address UAV–bird classification under low-SNR conditions, this paper proposes a classification framework based on VMD and STFT feature representations with weighted Gaussian Naive Bayes. The main contributions are summarized as follows:

(1) We propose a two-domain micro-Doppler feature extraction framework based on VMD modal decomposition and STFT subband statistics. The framework characterizes target micro-motion from both the modal and time–frequency domains under low-SNR conditions.
(2) We propose a weighted Gaussian Naive Bayes classification method based on the selected VMD and STFT features. Feature weights are estimated from the training data to reflect the relative contribution of individual feature components and support probabilistic UAV–bird classification.

Accordingly, the rest of this paper is organized as follows. Section 2 states the related work; Section 3 presents the proposed UAV–bird classification method; Section 4 presents the experimental results and analysis; and Section 5 concludes the paper.

## 2. Related Work

Traditional radar systems may provide insufficient resolution for detecting small UAVs with low radar cross sections. Micro-Doppler radar techniques can improve target recognition by capturing the micro-Doppler frequency shifts generated by moving components such as UAV rotors and propellers. Its core technologies include multi-dimensional feature extraction, high-resolution detection, and anti-interference optimization. Du et al. [9] proposed a noise-robust classification method based on micro-Doppler time–frequency features, in which denoising preprocessing and an SVM classifier were employed for ground moving target recognition. Amiri et al. [10] investigated STFT and GLCT representations for micro-Doppler-based target classification, demonstrating the importance of appropriate time–frequency representation for target discrimination. Kim and Ling [11] demonstrated that micro-Doppler signatures combined with a support vector machine can be used for activity classification, providing an early example of learning-based recognition from micro-Doppler features. Zhao et al. [12] proposed an adaptive weighted dual-input self-attention fusion method for dual-band early-warning radar micro-motion recognition, in which time–frequency information from different radar bands was jointly exploited for target recognition.

With the development of learning-based methods, convolutional neural networks and other deep models have also been introduced into radar target recognition. Chen et al. [13] used multistatic micro-Doppler signatures with a deep convolutional neural network for personnel recognition and gait classification. Zhao and Huang [14] developed a target recognition method for multi-aspect SAR images using EfficientNet and BiGRU. These studies demonstrate the potential of deep learning for extracting discriminative radar features. For UAV–bird recognition, Rahman et al. [15] analyzed radar micro-Doppler signatures of drones and birds at K-band and W-band. Yoon S et al. [16] proposed a FMCW radar-CNN theoretical framework, which mainly relies on learning micro-Doppler features to accurately identify multiple UAVs and birds. More recently, Zhang et al. [17] proposed an L/K dual-band micro-Doppler classification framework based on the Mamba state-space model, in which complementary information from different radar bands is exploited for UAV–bird discrimination, demonstrating the potential of sequence modeling for micro-Doppler target recognition. Song et al. [18] investigated deep-learning-based target detection and recognition using radar echo signals. Zheng et al. [19] investigated UAV target classification using micro-Doppler characteristics and machine-learning methods. Li et al. [20] proposed a short–medium–long multi-scale dynamic classification mechanism of “fast comprehensive multi-feature, sequential decision based on multiple likelihoods, and long-term precise segmentation of multiple factors”. In addition, Jiang et al. [21] developed a multi-dimensional feature fusion framework for small-UAV recognition using high-resolution radar, combining geometric structural information with micro-motion features derived from measured radar data. This work further indicates the value of integrating complementary feature representations for radar target recognition. Molchanov et al. [22] extracted low-dimensional features from aligned micro-Doppler signatures and validated small-UAV and bird classification using real X-band radar measurements.

Recent studies have also investigated low-SNR radar recognition and micro-Doppler feature enhancement. Wang et al. [23] studied radar-based classification of multirotor UAVs and birds under low-SNR conditions. Ma and Chen [24] proposed a one-dimensional cascaded denoising and classification framework for micro-Doppler-based radar target recognition. Yan et al. [25] investigated radar micro-Doppler signatures for drone-type recognition. Dong et al. [26] developed a variational time–frequency mode-tracking method for micro-Doppler signature extraction. Chen et al. [27] presented the LSS-FMCWR-1.0 dataset and a high-resolution micromotion feature extraction method for LSS targets.

Overall, existing UAVs and bird radar recognition methods can be broadly divided into handcrafted micro-Doppler feature methods, time–frequency or signal-decomposition-based methods, and data-driven learning methods. Handcrafted and single-domain features provide relatively interpretable target descriptions, but their discriminative characteristics may become less distinct when the micro-motion components are severely contaminated by noise. Deep learning and sequence-modeling approaches provide strong feature-learning capabilities, but they often involve larger training requirements and higher model complexity. Meanwhile, existing feature-fusion studies demonstrate the benefit of combining complementary target representations, but effective low-SNR characterization using compact and interpretable features remains an important issue. Therefore, this study constructs complementary feature representations from the VMD modal domain and the STFT time–frequency subband domain. Candidate features are evaluated using the training data, and the modal energy ratio and subband mean are retained and fused for subsequent weighted Gaussian Naive Bayes classification.

## 3. Methods

### 3.1. Analysis on Radar Echo Characteristics of Birds and UAVs in Low Signal-to-Noise Ratio Environments

In practical low-altitude surveillance scenarios, radar echoes from rotary-wing unmanned aerial vehicles (UAVs) and birds are jointly affected by various factors, including target motion, propagation distance, receiver noise, and environmental interference. As the target-to-radar range increases or the ambient noise level rises, the effective micro-motion information contained in the received echo becomes progressively masked by noise, resulting in a reduction in the signal-to-noise ratio (SNR). For rotary-wing UAVs, the periodic rotation of the rotors introduces additional micro-Doppler modulation into the radar echo on top of the Doppler shift induced by the translational motion of the target body [28]. Similarly, the periodic flapping motion of a bird’s wings produces characteristic micro-Doppler signatures in the received radar signal. Owing to the fundamentally different motion mechanisms of these two types of targets, their micro-motion signatures exhibit distinct characteristics in terms of temporal dynamics and frequency distribution. Under relatively high-SNR conditions, these differences can be clearly manifested in the time–frequency representation of the radar echoes. However, as the SNR decreases, the observability of the micro-motion components is progressively degraded by noise. Consequently, their periodicity and localized frequency structures become increasingly indistinct, thereby making target classification based on micro-Doppler information more challenging.

Considering that the classification method proposed in this study primarily exploits the micro-motion information induced by the internal motion of the targets, rather than relying on the total energy of the complete target echo, the power ratio between the micro-motion component and the noise component is adopted to define the SNR for the low-SNR experiments. The micro-motion SNR is defined by Formula (1).

(1) $${\mathrm{SNR}}_{m}=10\log_{10}\left(\frac{P_{m}}{P_{n}}\right)$$

where $P_{m}$ and $P_{n}$ denote the average powers of the target micro-motion component and the noise component, respectively. For rotary-wing UAVs, $P_{m}$ corresponds to the power of the micro-motion component induced by the periodic rotation of the rotors, whereas for birds, it represents the power of the micro-motion component associated with periodic wing flapping.

To distinguish the micro-motion SNR from the SNR of the complete target echo, the overall SNR of the complete target echo is further defined by Formula (2).

(2) $${\mathrm{SNR}}_{t}=10\log_{10}\left(\frac{P_{t}}{P_{n}}\right)$$

where $P_{t}$ denotes the average power of the complete target echo, including both the translational motion component of the target body and the internal micro-motion component. Because the echo powers associated with the translational and micro-motion components are generally different, the two SNR definitions are not necessarily equivalent. Since the classification procedure in this study primarily focuses on the micro-Doppler information generated by internal motions, such as rotor rotation and wing flapping, the SNR referred to hereafter denotes the micro-motion SNR unless otherwise specified.

In the present simulation, pulse compression is not explicitly modeled. The SNR used for the subsequent classification experiments is therefore defined on the micro-motion slow-time echo sequence immediately before the VMD/STFT processing stage. Specifically, the reported SNR refers to the power ratio between the extracted micro-motion component and the additive noise component.

Based on the observability of micro-motion structures under different SNR conditions, the experimental conditions are further divided into several SNR regimes. When ${\mathrm{SNR}}_{m}>-5\;\mathrm{dB}$, the micro-motion modulation structures are generally well-preserved in the received echo, and this range is defined as the normal-SNR regime. When $-10\;\mathrm{dB}<{\mathrm{SNR}}_{m}\leq -5\;\mathrm{dB}$, the influence of noise on the micro-motion structures becomes more pronounced, resulting in reduced observability of the internal motion information; this range is therefore defined as the low-SNR regime. Furthermore, when ${\mathrm{SNR}}_{m}\leq -10\;\mathrm{dB}$, the micro-motion signal is subject to severe noise masking and is consequently defined as the extremely low-SNR regime. These definitions are adopted as the unified criteria for the subsequent low-SNR experiments and performance evaluations.

In practical radar systems, the received signal is typically represented as a complex baseband echo, which can be expressed by Formula (3).

(3) $$s_{c}(t)=I(t)+jQ(t)$$

where  I(t) and Q(t) denote the in-phase and quadrature components, respectively. Since the variational mode decomposition (VMD) employed in this study operates on real-valued sequences, the complex baseband echo is converted into a real-valued slow-time sequence prior to modal decomposition, which can be expressed by Formula (4).

(4) x(t)=Re{s(t)}=sI(t)

All subsequent VMD operations are performed on the real-valued signal s(t). This preprocessing step satisfies the input requirements of VMD and provides a consistent signal representation for modal decomposition and feature extraction under different SNR conditions. To maintain consistency throughout the signal processing pipeline, the subsequent short-time Fourier transform (STFT) analysis also uses the same real-valued slow-time signal as its input. Based on the aforementioned SNR definition and signal-processing convention, the time-domain characteristics of the target echoes under different SNR conditions are further investigated.

Figure 1 shows the time-domain waveforms of quadrotor UAV echoes at SNRs of 10, 0, −5, and −10 dB. At relatively high SNRs, periodic fluctuations associated with rotor-induced micro-motion are clearly observable. As the SNR decreases, noise progressively masks the micro-motion components, and the periodic structure becomes less distinguishable. When SNR≤−5 dB, the waveform exhibits pronounced randomness, making the rotor-induced micro-motion difficult to identify directly in the time domain. Figure 2 presents the time-domain waveforms of bird echoes under the same SNR conditions. Compared to UAV echoes, bird echoes exhibit relatively low-frequency fluctuations associated with wing-flapping motion. As the SNR decreases, these periodic characteristics are progressively obscured by noise and become difficult to distinguish when SNR≤−5 dB. Figure 3 compares the time-domain waveforms of pure UAV echoes and mixed UAV–bird echoes. At relatively high SNRs, differences between the two scenarios are observable, whereas these differences become increasingly indistinguishable as the SNR decreases. These results indicate that direct time-domain discrimination is substantially degraded under low-SNR conditions, motivating the use of modal and localized time–frequency features for subsequent target classification.

In summary, under low-SNR conditions, the micro-Doppler modulation patterns generated by UAV rotor rotation and bird wing flapping are both severely affected by noise, which reduces their observability in the time domain. However, because the two target types have fundamentally different motion mechanisms, differences can still be observed in their frequency-component distributions, modal energy distributions, and local time–frequency characteristics. Therefore, improving target discrimination under low-SNR conditions requires more than a single representation in either the time domain or the frequency domain. In this study, radar echoes are characterized from two complementary perspectives. The first focuses on the modal characteristics obtained through signal decomposition, while the second describes the statistical characteristics of local time–frequency regions. These two types of features are then fused to provide complementary information for UAV and bird classification under low-SNR conditions.

### 3.2. Overall Research Idea and Methodological Framework

To address the degradation of micro-Doppler structures and the reduced observability of target micro-motion information under low-SNR conditions, as analyzed in Section 3.1, this study proposes a UAV and bird classification method based on the fusion of variational mode decomposition (VMD) and time–frequency subband statistical features. The proposed method characterizes the micro-motion information of the targets at different feature scales by separately extracting VMD-based modal features and STFT-based time–frequency subband statistical features. These features are subsequently processed through candidate feature construction, feature selection, and probabilistic statistical classification to achieve target identification. The overall framework of the proposed method is illustrated in Figure 4 and mainly consists of three stages: radar echo preprocessing, two-scale feature extraction, and candidate feature construction and classification decision-making.

As illustrated in Figure 4, the proposed method mainly consists of the following three stages.

(1) Radar Echo Input and Preprocessing

First, radar echo signals from UAV and bird targets are acquired and preprocessed to reduce the effects of the DC component and noise interference on subsequent feature extraction. For complex baseband echoes, the real-valued slow-time signal described in Section 3.1 is obtained through the corresponding signal processing procedure. This signal is then used as the common input for feature extraction based on VMD and STFT.

(2) Two-Scale Feature Extraction

In this stage, complementary micro-motion features are extracted from different representations of the radar echo signal. First, variational mode decomposition (VMD) is applied to the preprocessed signal to obtain multiple intrinsic mode components. Statistical features are then calculated for these components to characterize the energy distribution and structural properties of the target micro-motion signal across different modes. Subsequently, the short-time Fourier transform (STFT) is applied to obtain the time–frequency representation of the target micro-Doppler signal. The resulting spectrum is divided into multiple frequency subbands, and statistical features, including the mean, standard deviation, and entropy, are extracted from each subband. These features characterize the local distribution and temporal variation in micro-Doppler energy in the time–frequency domain. The two feature extraction procedures characterize the target micro-motion information from the perspective of modal characteristics and time–frequency statistical characteristics, respectively, thereby forming a candidate feature set for subsequent feature selection.

(3) Candidate Feature Construction, Feature Selection, and Bayesian Classification

After the two feature extraction procedures, the VMD modal features and the STFT time–frequency subband statistical features are combined to construct the candidate feature set. Considering the differences in the discriminative capability of the extracted features and the potential redundancy among them, the candidate features are further evaluated and selected to retain the most informative features. The selected features are then normalized. Based on the training data, the relative weights of the selected features are determined to construct the final feature representation, which consists of two selected feature families. The resulting feature representation is subsequently input into a Gaussian Naive Bayes classifier. The classifier establishes the conditional probability distributions of the selected features for different target classes and applies the maximum a posteriori (MAP) criterion to obtain the final classification decision for UAV and bird targets.

To provide a more intuitive representation of the implementation procedure, the complete target recognition process is further illustrated in Figure 5 based on the overall methodological framework.

As illustrated in Figure 5, the preprocessed radar echoes are fed into two parallel feature extraction branches based on VMD and STFT, respectively. Three types of candidate features are extracted from each branch, resulting in a candidate feature set consisting of six feature types. The candidate features are then subjected to unified processing and feature evaluation, from which the selected feature families are used to construct the final fused feature vector for subsequent classification. For the selected features, normalization is first performed using the statistical parameters obtained from the training set to eliminate the effects caused by differences in feature scales. Subsequently, feature discriminative weights are determined based on the training data, and Gaussian conditional probability models are established for each target class. For an unknown sample, the weighted posterior probabilities under different target classes are calculated, and the final classification result is determined according to the maximum a posteriori (MAP) criterion, thereby enabling UAV and bird identification.

In this framework, the two feature extraction branches provide complementary descriptions of target micro-motion characteristics from the modal and time–frequency subband perspectives. The subsequent feature evaluation and selection process reduces redundant information while retaining the most discriminative characteristics for classification. Feature normalization and discriminative weighting further improve the consistency and contribution balance among different feature dimensions. On this basis, Gaussian conditional probability modeling provides a probabilistic representation of the selected feature distributions, and the MAP criterion is employed to determine the target class. The detailed extraction procedures of the VMD and STFT-based branches, together with the construction and selection of the six candidate feature types, are presented in Section 3.3.

To further clarify the specific implementation process of each step, this paper provides the corresponding algorithm description, as shown in Algorithm 1.
**Algorithm 1:** VMD and STFT Feature Extraction, Feature Selection, and Weighted Gaussian Naive Bayes ClassificationInput: Radar echo signal $x\left(t\right)$, training labels Y, VMD parameters K and α, STFT parameters $N_{w}$, $N_{o}$, and $N_{FFT}$, and the number of STFT subbands L. Output: Predicted class label $\hat{y}$ 1:    Partition the samples of each Monte Carlo trial into ten folds       before any data-dependent processing. 2:    for each cross-validation fold do 3:           VMD Feature Extraction 4:           Apply VMD to the radar echo using the predefined parameters. 5:           Extract the modal energy ratio (MER), frequency discrete              entropy (FDE), and singular value ratio (SVR). 6:           STFT Feature Extraction 7:           Compute the STFT using the predefined window and FFT parameters. 8:           Retain the positive-frequency spectrum and divide it into              L equal-width subbands. 9:           Extract the subband mean, standard deviation, and entropy. 10:         Candidate Feature Selection 11:         Construct the six candidate feature families from the VMD              and STFT branches. 12:         Evaluate their discriminative capabilities using the              training fold only. 13:         Retain the VMD modal energy ratio and STFT subband mean              feature families. 14:         Feature Normalization and Fusion 15:         Estimate normalization statistics from the training fold only. 16:         Normalize the training and test features using the              training-fold statistics. 17:         Concatenate the selected MER and subband mean features to              form the final fused feature vector with dimension d=K+L. 18:         Feature Weight Estimation 19:         Compute the discriminative score of each numerical feature              component using the training fold. 20:         Normalize the discriminative scores to obtain the feature              Weights $w_{j},\;j\;=\;1,\ldots ,d$. 21:         Keep the estimated weights fixed for the corresponding              test fold. 22:         Weighted Gaussian Naive Bayes Training 23:         Estimate the class prior probabilities from the training fold. 24:         Estimate the class-conditional Gaussian parameters for each              feature component. 25:         Classification 26:         for each test sample do 27:               Compute the weighted class-conditional probabilities                    for all candidate classes. 28:               Calculate the corresponding posterior probabilities. 29:               Assign the class with the maximum posterior probability. 30:         end for 31: end for 32: Return the predicted class labels $y_{hat}$.

### 3.3. Two-Scale Feature Extraction

To characterize the target micro-motion information under low-SNR conditions, this study extracts complementary features from two distinct representation scales of the radar echo. Specifically, variational mode decomposition (VMD) is employed to characterize the modal structure and energy distribution of the micro-motion signal, while the short-time Fourier transform (STFT) is used to characterize the localized time–frequency distribution of micro-Doppler energy. The extracted VMD modal features and STFT-based time–frequency subband statistical features are subsequently combined to construct the candidate feature set for subsequent feature selection and classification. The overall two-scale feature extraction process is illustrated in Figure 6.

Specifically, the preprocessed radar echo signals are independently processed by the two feature extraction branches shown in Figure 6. In branch (a), variational mode decomposition (VMD) is first applied to adaptively decompose the radar echo into *K* intrinsic mode functions (IMFs) with different center frequencies. Based on the resulting modal components, three modal-domain features, namely the modal energy ratio, frequency discrete entropy, and singular value ratio, are extracted to characterize the energy distribution and structural characteristics of the target micro-motion across different modes. In branch (b), the radar echo is transformed into a time–frequency representation using the short-time Fourier transform (STFT). The positive-frequency component of the resulting micro-Doppler spectrum is retained and uniformly divided into *L* subbands with equal frequency bandwidth. For each subband, three statistical descriptors, including the subband mean, subband standard deviation, and subband entropy, are calculated to characterize the local distribution and variation in micro-Doppler energy in the time–frequency domain.

Accordingly, the two branches provide complementary descriptions of the target micro-motion from the modal and time–frequency perspectives, respectively. The VMD branch yields three modal domain candidate feature types, while the STFT branch provides three time–frequency subband statistical feature types, resulting in six candidate feature types in total. The subsequent evaluation and selection of these candidate features are presented separately in Section 3.3.3.

#### 3.3.1. VMD-Based Modal Feature Extraction

In the proposed feature fusion framework, the radar echo signal is first preprocessed and decomposed using variational mode decomposition (VMD). Under low-SNR conditions, noise can mask the micro-motion components, making their frequency-domain characteristics difficult to observe directly. VMD is therefore used to decompose the radar echo into multiple band-limited modal components. This provides a structured representation of micro-motion information at different frequency scales. VMD is an adaptive signal decomposition method based on variational optimization. It decomposes a signal into several intrinsic mode functions (IMFs) with limited bandwidth. Unlike methods that rely on predefined basis functions, VMD adaptively determines the frequency bands according to the signal characteristics. It also provides adaptive frequency decomposition capability under noisy conditions. For rotary-wing UAVs, the periodic rotation of the blades produces a broad micro-Doppler spectrum with multiple frequency components. In comparison, bird echoes usually have a more concentrated spectral distribution because of their relatively low wing-beating frequency. Based on this difference, VMD is applied to the radar echo to obtain multiple modal components. Statistical features are then extracted from each mode to describe the spectral characteristics of UAV and bird echoes. The radar echo signal s(t) is defined by Formula (5) and decomposed into K modal components using VMD.

(5) $$s(t)=\sum_{k=1}^{K}u_{k}(t)$$

where $u_{k}(t)$ denotes the *k*-th modal component with a corresponding central frequency of $\omega_{k}$.

To ensure the reproducibility and consistency of the VMD decomposition, the principal parameters were fixed throughout all quantitative experiments. The number of modes, quadratic penalty factor, and absolute convergence tolerance were set to  K=8, α=2000, and $10^{-7}$, respectively, while the default peak-based center-frequency initialization strategy of the MATLAB 2022b VMD implementation was adopted. The parameter K controls the decomposition granularity, whereas α regulates the bandwidth of the individual modes. Too few modes may merge components with different frequency characteristics, whereas too many modes may introduce redundant components and increase computational complexity. The same parameter configuration was used for all SNR levels and noise conditions to ensure consistent and comparable decomposition results.

To provide an intuitive illustration of the VMD decomposition process, Figure 7 presents a representative example with four modes selected from the eight modes obtained through decomposition. The radar echo signal is adaptively decomposed into several intrinsic mode functions (IMFs) with different center frequencies. These modes represent motion components at different frequency scales. The modes with higher center frequencies mainly capture rapid micro-motion variations, whereas those with lower center frequencies primarily represent relatively slow motion components. This decomposition provides a structured representation of the radar echo signal and establishes a basis for subsequent statistical feature extraction.

Based on the decomposed IMFs, three candidate feature families are extracted to characterize the modal properties. They are the modal energy ratio, frequency discrete entropy, and singular value ratio. The modal energy ratio describes the energy distribution across different modes. The frequency discrete entropy measures the dispersion of the modal center frequencies. The singular value ratio reflects the structural concentration of the decomposed signal. These features are combined with the STFT-derived features to form the candidate feature space for subsequent feature selection and classification.

First, the energy of each mode is defined by Formula (6).

(6) $$E_{k}=\sum_{n}|u_{k}(n){|}^{2}$$

The normalized energy ratio is defined by Formula (7).

(7) $$P_{k}=\frac{E_{k}}{\sum_{k=1}^{K}E_{k}}$$

By treating energy distribution as a probability distribution, the frequency discrete entropy can be defined by Formula (8).

(8) $$Q_{k}=-\sum_{k=1}^{K}P_{k}\log P_{k}$$

Meanwhile, a matrix $U=[u_{1},u_{2},\cdots ,u_{K}]$ is constructed from the signals of all modes, and singular value decomposition is performed according to Formula (9).

(9) $$U=A\sum B^{T}$$

Here, $\Sigma =\mathrm{diag}(\sigma_{1},\sigma_{2},\cdots ,\sigma_{K})$ denotes the singular value matrix, and the corresponding singular value ratio is defined by Formula (10).

(10) $$\lambda_{k}=\frac{\sigma_{k}}{\sum_{k=1}^{K}\sigma_{k}}$$

The modal energy ratio reflects the distribution of signal energy across different modes. Rotary-wing UAVs usually have a wide micro-Doppler frequency range, so their signal energy is distributed across multiple modes. For birds, the energy is mainly concentrated in a few modes. This difference provides useful information for target discrimination. Frequency discrete entropy measures the dispersion of energy among different frequency components. Rotary-wing UAVs generally have higher entropy values because their echoes contain multiple frequency components. Bird echoes usually have lower entropy values because their frequency components are more concentrated. The singular value ratio describes the structural characteristics of the decomposed signal. The complex micro-motion of Rotary-wing UAVs usually produces multiple significant singular values. Bird echoes are generally dominated by a few singular values. Therefore, the two target types show different singular value distributions. Therefore, the VMD-based feature vector can be expressed by Formula (11).

(11) $$F_{vmd}=\left[P_{1},\;\ldots ,\;P_{k},\;Q_{1},\;\ldots ,\;Q_{k},\;\lambda_{1},\;\ldots ,\;\lambda_{k}\right]$$

However, the frequency domain features obtained using VMD alone cannot fully describe the temporal evolution of target micro-motion. To characterize the time-varying micro-Doppler patterns of rotary-wing UAVs and birds, STFT is further applied to analyze the signal in the time–frequency domain. The resulting features provide information that complements the VMD features. By combining the two types of features, the micro-motion characteristics of the targets can be described more comprehensively.

#### 3.3.2. Feature Extraction Based on Time–Frequency Spectrum Segmentation

When the signal-to-noise ratio is low, the micro-Doppler features are not obvious in either the time domain or the frequency domain. Through time–frequency analysis, the local energy distribution characteristics can be enhanced in the joint time–frequency domain. The short-time Fourier transform (STFT) is adopted to perform time–frequency analysis on the radar echo signal, and the corresponding STFT time–frequency spectrum is denoted as $S\left(t,\;f\right)$. Rotary-wing UAVs and bird targets exhibit different energy distribution characteristics in the time–frequency domain. Due to the approximately periodic structure of the modulation induced by the rotation of the UAV blades, the positive-frequency component of the time–frequency spectrum can effectively characterize the differences in the micro-Doppler features of the target.

Therefore, only the positive-frequency component of the time–frequency spectrum is selected and divided into L equal-width subbands along the frequency axis. The overall process of time–frequency spectrum segmentation and subband feature extraction is illustrated in Figure 6b. As shown in the figure, the radar echo signal is first transformed into the STFT time–frequency spectrum, from which only the positive frequency portion is retained. This portion is then uniformly divided into L equal-width subbands, where the k-th subband is denoted by $\;B_{k}$ and contains N time–frequency units. Feature vectors are constructed based on the statistical distribution of each subband. On this basis, subband statistical features are extracted from two aspects: energy distribution and statistical fluctuation characteristics, mainly including subband mean, subband standard deviation, and subband entropy, which are then used to construct feature vectors for subsequent analysis.

To ensure reproducibility, the STFT parameters were kept fixed throughout all experiments. A Hamming window with a length of 256 samples was used, with an overlap of 50% between adjacent windows. The FFT size was set to 512 points. For the resulting two-sided time–frequency spectrum, only the positive-frequency component was retained for feature extraction. Because the STFT was applied to a real-valued slow-time signal, the positive and negative frequency components exhibit conjugate symmetry. Therefore, the negative frequency component was removed to avoid redundant spectral information and reduce the feature dimension. The retained positive frequency range was uniformly divided into image subbands along the frequency axis, with each subband having the same bandwidth. Three statistical features were calculated for each subband: the subband mean, subband standard deviation, and subband entropy. The subband mean describes the average energy level within the corresponding frequency region. The subband standard deviation characterizes fluctuations in the energy distribution, while the subband entropy reflects the statistical complexity of the local frequency region. All STFT parameters were kept unchanged throughout the experiments, including the window type, window length, overlap ratio, FFT size, and number of subbands. These parameters were not adjusted according to the SNR level or the test dataset.

First, the mean value of each subband is defined by Formula (12).

(12) $$\mu_{k}=\frac{1}{N_{k}}\sum_{\left(t,f\right)\in S_{k}}S(t,f)$$

The feature vector $\mu =[\mu_{1},\mu_{2},\ldots ,\mu_{k}]$ is constructed from the mean values of all subbands, which characterizes the average energy distribution across different frequency bands. The standard deviation of each subband is defined by Formula (13).

(13) $$\sigma_{k}=\sqrt{\frac{1}{N_{k}}\sum_{\left(t,f\right)\in S_{k}}{\left(S(t,f)-\mu_{k}\right)}^{2}}\;$$

The standard deviations of each subband form a feature vector $\sigma =[\sigma_{1},\sigma_{2},\ldots ,\sigma_{K}]$ that describes the degree of energy fluctuation within each frequency band.

By treating the amplitude distribution within a subband as a probability distribution, the subband entropy is defined by Formula (14).

(14) $$H_{k}=-\sum_{i}p_{k,i}logp_{k,i}$$

where $p_{k,i}$ denotes the probability of the i-th amplitude value in the k-th subband.

An entropy feature vector $H=[H_{1},H_{2},\ldots ,H_{K}]$ is constructed to measure the information complexity within each subband. Similarly, the time–frequency statistical feature vector is defined by Formula (15).

(15) $$F_{tf}=\left[\mu_{1},\;\ldots ,\;\mu_{K},\;\sigma_{1},\;\ldots ,\;\sigma_{K},\;H_{1},\;\ldots ,\;H_{K}\right]$$

Rotary-wing UAVs usually exhibit continuous micro-Doppler modulation caused by periodic rotor rotation, resulting in energy distribution across multiple frequency subbands. In comparison, bird targets generally show relatively concentrated micro-Doppler characteristics due to wing-flapping motion, with energy mainly distributed in several low-frequency regions. Therefore, differences in subband energy distribution provide useful information for target discrimination. The subband mean features characterize the energy distribution of different targets in the time–frequency domain. Due to rotor-induced periodic modulation, UAV echoes usually contain effective micro-Doppler components over a wider frequency range, leading to more diverse energy distributions among subbands. In contrast, bird echoes tend to exhibit more concentrated energy patterns, especially when high-frequency components are dominated by noise. The subband standard deviation reflects the variation in spectral energy within each frequency region. Subbands containing clear micro-Doppler structures usually exhibit larger variations, while noise-dominated regions tend to show relatively stable or irregular distributions. Meanwhile, entropy features describe the complexity of the spectral energy distribution. Subbands with richer micro-Doppler components generally present more complex energy distributions and higher entropy values, whereas noise-dominated subbands exhibit weaker structural information.

Overall, UAV and bird targets show different distribution characteristics in the STFT subband statistical feature space. However, individual feature dimensions only provide partial descriptions of target characteristics and cannot directly form reliable classification decisions. Therefore, these complementary features are further integrated with VMD-based features to construct a unified feature representation for subsequent probabilistic classification.

#### 3.3.3. Candidate Feature Construction and Feature Selection

Following the two feature extraction branches described above, candidate features are constructed from the VMD modal domain and the STFT time–frequency subband domain. The VMD branch provides three candidate feature families: the modal energy ratio (MER), frequency discrete entropy (FDE), and singular value ratio (SVR). These features describe the modal energy distribution, frequency complexity, and structural characteristics of the target micro-motion, respectively.

The STFT branch provides three additional candidate feature families: the subband mean, subband standard deviation, and subband entropy. These features characterize the local energy distribution, fluctuation, and complexity of the micro-Doppler spectrum within different frequency subbands. The complete candidate feature set is defined by Formula (16).

(16) $$F_{cand}=\left[F_{\mathrm{MER}},F_{\mathrm{FDE}},F_{\mathrm{SVR}},F_{\mathrm{Mean}},F_{Std},F_{\mathrm{Ent}}\right]$$

where $F_{\mathrm{MER}}$, $F_{\mathrm{FDE}}$ and $F_{\mathrm{SVR}}$ denote the three VMD-based feature families, while $F_{\mathrm{Mean}}$, $F_{Std}$ and $F_{\mathrm{Ent}}$ denote the three STFT-based feature families. The six candidate feature families are not used directly as the final classifier input. Their discriminative capabilities may differ, and some feature families may contain redundant information. Therefore, feature selection is performed using the training fold only. The candidate feature families are evaluated according to their classification performance and complementary characteristics. The VMD-based and STFT-based features are first assessed within their respective representation domains, followed by cross-domain evaluation of the retained candidates.

Based on this selection procedure, the VMD modal energy ratio and the STFT subband mean are retained as the final feature families. The modal energy ratio describes how micro-motion energy is distributed among the VMD modes, whereas the subband mean describes the average micro-Doppler energy within different STFT frequency regions. These two feature families therefore provide complementary descriptions of the target micro-motion characteristics.

The final fused feature vector is defined by Formula (17).

(17) $$F={\left[\begin{array}{cc} F_{\mathrm{MER}}^{T} & F_{\mathrm{Mean}}^{T} \end{array}\right]}^{T}$$

where $F_{\mathrm{MER}}\in R^{K},F_{\mathrm{Mean}}\in R^{L}$. Accordingly, the dimensionality of the final feature vector is given by Formula (18).

(18) d=K+L

Here, K denotes the number of VMD modes and  L denotes the number of STFT frequency subbands. Thus, the term “two final feature families” refers to two selected feature sources rather than two numerical dimensions. All subsequent normalization, feature-weight estimation, and weighted GNB classifier are performed on this d-dimensional fused feature vector.

### 3.4. Feature Fusion and Weighted Gaussian Naive Bayes Classification

After candidate feature construction and selection, the VMD modal energy ratio and the STFT subband mean are retained for subsequent classification. The two feature families are defined by Formula (19).

(19) $$\begin{array}{ll} F_{\mathrm{MER}} & ={\left[f_{\mathrm{MER},1},f_{\mathrm{MER},2},\ldots ,f_{\mathrm{MER},K}\right]}^{T},\\ F_{\mathrm{Mean}} & ={\left[f_{\mathrm{Mean},1},f_{\mathrm{Mean},2},\ldots ,f_{\mathrm{Mean},L}\right]}^{T} \end{array}$$

where K denotes the number of VMD modes and L denotes the number of STFT frequency subbands. Since the two feature families have different numerical ranges and statistical scales, standardization is applied before feature fusion. Let $F_{j}$ denote the j-th numerical feature component. The standardized feature is defined by Formula (20).

(20) $${\hat{F}}_{j}=\frac{F_{j}-\mu_{j}^{\mathrm{tr}}}{\sigma_{j}^{\mathrm{tr}}+\varepsilon },j=1,2,\ldots ,d$$

where $\mu_{j}^{\mathrm{tr}}$ and $\sigma_{j}^{\mathrm{tr}}$ are the mean and standard deviation of th j-th feature component estimated from the current training fold. The constant ε is a small positive value introduced to avoid division by zero. The normalization parameters are estimated using the training fold only and are then applied to the corresponding test fold.

After normalization, the selected VMD and STFT features are concatenated to form the final d-dimensional fused feature vector, as defined by Formula (21).

(21) $$\hat{F}={\left[\begin{array}{cc} {\hat{F}}_{\mathrm{MER}}^{T} & {\hat{F}}_{\mathrm{Mean}}^{T} \end{array}\right]}^{T}$$

where d=K+L, as defined in Section 3.3.3. The fused representation contains the modal energy distribution from the VMD domain and the local energy distribution from the STFT subband domain. These two types of information provide complementary descriptions of the target micro-motion characteristics.

Different numerical feature components may contribute differently to UAV and bird discrimination. Therefore, a discriminative score is estimated for each feature component using the training samples. For the j-th feature component, the score is defined by Formula (22).

(22) $$D_{j}=\frac{{\left(\mu_{j,U}-\mu_{j,B}\right)}^{2}}{\sigma_{j,U}^{2}+\sigma_{j,B}^{2}+\varepsilon },j=1,2,\ldots ,d$$

where $\mu_{j,U}$ and $\mu_{j,B}$ are the class means of the UAV and bird samples, respectively. The terms $\sigma_{j,U}^{2}$ and $\sigma_{j,B}^{2}$ denote the corresponding within-class variances. A larger $D_{j}$ indicates greater inter-class separation and lower within-class variation. The feature weights are defined by Formula (23), where the discriminative scores are normalized across all feature components.

(23) $$w_{j}=\frac{D_{j}}{\sum_{r=1}^{d}D_{r}},j=1,2,\ldots ,d$$

The weights are subject to the constraint given by Formula (24).

(24) $$\sum_{j=1}^{d}w_{j}=1$$

The resulting weight $w_{j}$ represents the relative contribution of the j-th numerical feature component in the subsequent weighted Gaussian Naive Bayes classifier. All class statistics and feature weights are estimated exclusively from the current training fold and remain fixed when classifying the corresponding test fold. Thus, the test samples are not involved in the estimation of normalization parameters, feature weights, or classifier parameters.

Based on the two-scale feature extraction and feature selection described in Section 3.3, the retained VMD modal energy ratio and STFT subband mean features are normalized and fused to construct the final feature representation. The overall procedure of feature selection, normalization, fusion, and weighted Gaussian Naive Bayes classification is illustrated in Figure 8.

As shown in Figure 8, the proposed framework consists of three main stages: (1) Candidate Feature Input, (2) Feature Selection and Fusion, and (3) Classification and Output. In stage (1), three VMD-based candidate feature families, including the modal energy ratio (MER), frequency discrete entropy (FDE), and singular value ratio (SVR), together with three STFT-based candidate feature families, including the subband mean, subband standard deviation, and subband entropy, are constructed to form the initial candidate feature space. In stage (2), the discriminative capabilities and complementary characteristics of the candidate feature families are evaluated using the training data. Based on the feature-selection results, the VMD modal energy ratio and STFT subband mean are retained as the final feature families. The selected features are subsequently normalized using statistical parameters estimated exclusively from the training set and concatenated to construct the final fused feature representation. In stage (3), feature weights are determined from the training data, and the fused feature vector is fed into the weighted Gaussian Naive Bayes classifier. The posterior probabilities of the target classes are calculated, and the final UAV–bird classification result is obtained according to the maximum a posteriori (MAP) criterion.

Based on the fused feature vector $\hat{F}$ define above, a weighted Gaussian Naive Bayes classifier is constructed for UAV–bird discrimination. Each feature component is modeled by a class-conditional Gaussian distribution, while the feature weights obtained from the training fold are incorporated into the probabilistic decision process. The category set is defined by Formula (25).

(25) $$C=\left\{C_{1},C_{2}\right\}$$

where $C_{1}$ and $C_{2}$ represent the rotorcraft UAV and the bird target, respectively.

Within the framework of probabilistic inference, given a feature vector F, the posterior probability of class is defined by Formula (26).

(26) $$P(C_{i}|F)=\frac{P(F|C_{i})P(C_{i})}{P(F)}$$

Based on the maximum a posteriori (MAP) criterion, the classification result is determined by Formula (27).

(27) $$\hat{C}=\arg\max_{C_{i}}P(C_{i}|F)$$

By omitting the normalization terms that are independent of the class label, the corresponding decision function is given by Formula (28).

(28) $$\hat{C}=\arg\max_{C_{i}}P(F|C_{i})P(C_{i})$$

To characterize the statistical distribution of the features for different classes, conditional probability modeling is performed for each feature dimension. Assuming that each feature follows a Gaussian distribution under each class condition, the conditional probability density is defined by Formula (29).

(29) $$P(F_{j}|C_{i})=\frac{1}{\sqrt{2\pi \sigma_{ij}^{2}}}\exp\left(-\frac{{\left(F_{j}-\mu_{ij}\right)}^{2}}{2\sigma_{ij}^{2}}\right)$$

Under the conditional independence assumption of weighted Gaussian Naive Bayes, the joint class-conditional probability density of the selected fused feature vector is given by Formula (30).

(30) $$P(F|C_{i})=\prod_{j=1}^{6}P(F_{j}|C_{i})$$

Since the selected feature components may exhibit different discriminative capabilities, feature weights estimated from the training data are incorporated into the weighted Gaussian Naive Bayes model [26]. The weight determination procedure is described in the preceding subsection, and the resulting weights remain fixed during testing. The weighted class-conditional likelihood is defined by Formula (31).

(31) $$P(F|C_{i})=\prod_{j=1}^{6}{\left[P(F_{j}|C_{i})\right]}^{w_{j}},j=1,\ldots ,d$$

To avoid underflow in numerical computation, the model is transformed into the logarithmic domain, and the final decision function is given by Formula (32).

(32) $$\hat{C}=\arg\max_{C_{i}}\left[\ln P(C_{i})+\sum_{j=1}^{6}w_{j}\ln P(F_{j}|C_{i})\right]$$

This classification process can be represented as a typical Naive Bayes network structure, with its topological relationships illustrated in Figure 9.

As shown in Figure 9, the class variable C is the parent node of the d feature nodes $F_{1},F_{2},\ldots ,F_{d}$. No direct connections are introduced among the feature nodes, which reflects the conditional independence assumption of weighted Gaussian Naive Bayes. The class prior probabilities and class-conditional Gaussian parameters are estimated from the training data. The posterior probability of each class is then calculated, and the final class label is determined using the MAP criterion.

From a structural perspective, this framework decouples feature extraction from classification. The selected MER and subband mean features provide complementary statistical descriptions of target micro-motion, while the weighted GNB classifier provides a probabilistic decision framework for the fused feature space. Within the fused feature space, the weighted Gaussian Naive Bayes model characterizes the class-conditional distributions of UAV and bird samples and incorporates the relative contribution of each feature component. The final target label is obtained using the MAP criterion. This provides an interpretable probabilistic framework for UAV–bird discrimination under low-SNR conditions.

## 4. Experimentation and Analysis

### 4.1. Experimental Setup and Dataset Description

To evaluate the proposed method for UAV and bird classification, a unified experimental setting is established in terms of Dataset construction, radar parameters, signal-processing parameters, data partitioning, and evaluation metrics.

Throughout the experiments, SNR refers to the micro-motion signal-to-noise ratio $SNR_{m}$ defined in Section 3.1 unless otherwise specified. Conditions satisfying $-10\;\mathrm{dB}<SNR_{m}\leq -5\;\mathrm{dB}$, are referred to as low-SNR conditions, while $SNR_{m}\leq -10\;\mathrm{dB}$ is regarded as the extremely low-SNR regime. As $SNR_{m}$ decreases, the target micro-motion components are increasingly affected by noise, and the corresponding micro-Doppler structures become less distinguishable. This makes UAV and bird classification more challenging.

For a consistent comparison, all evaluated methods use the same radar parameters, data-partitioning strategy, training samples, and test samples. All data-dependent procedures, including feature normalization, feature selection, feature-weight estimation, and classifier-parameter estimation, are performed using the current training fold only. The parameters obtained from the training fold remain fixed when processing and classifying the corresponding test fold. The test samples are not involved in the estimation of these parameters.

#### 4.1.1. Simulation Dataset Generation

The experimental data were generated using scattering models for rotary-wing UAV and bird micro-motion targets. The UAV was modeled as a typical quadrotor. Its azimuth and pitch angles were sampled within predefined ranges, while the rotor speed was varied over multiple levels to represent different rotational states. Bird targets were generated using a wing-flapping motion model, with multiple wing-flapping frequencies considered to represent different motion states.

To evaluate the classification performance under different noise and interference conditions, a comprehensive simulation dataset was constructed over a wide SNR range. Throughout the experiments, SNR refers to the micro-motion signal-to-noise ratio $SNR_{m}$ defined in Section 3.1, namely, the ratio between the power of the micro-motion component generated by rotor rotation or wing flapping and the power of the additive noise component. For experiments involving clutter or impulse interference, these components were introduced as additional interference terms, while the definition of $SNR_{m}$ remained unchanged.

Three noise scenarios were considered:

(1) AWGN-only: standard additive white Gaussian noise was added as the baseline noise condition;

(2) Clutter-AWGN: compound-Gaussian ground clutter was superimposed with AWGN to evaluate the influence of clutter interference;

(3) Mixed-interference: AWGN, compound-Gaussian clutter, and 5% impulse noise were jointly introduced to represent a more challenging interference condition.

The evaluated SNR range was from −25 dB to 15 dB, covering both relatively high-SNR and severe low-SNR conditions.

At each SNR level, the dataset contained two target classes, namely UAVs and birds. The target-state combinations consisted of 11 azimuth-angle settings, 8 pitch-angle settings, and 6 motion-parameter settings corresponding to rotor speed or wing-flapping frequency. Therefore, the total number of samples generated in each trial was N = 2 × 11 × 8 × 6 = 1056. To reduce the influence of random noise realizations and improve the stability of the reported results, 50 independent Monte Carlo trials were conducted at each SNR level. The radar echoes and noise realizations were independently generated in each trial, and the subsequent training and test partitioning was also performed independently. The resulting simulation dataset provides a controlled experimental setting for evaluating the proposed method under different SNR and interference conditions.

The parameters governing the echo signal simulation for the rotary-wing UAV and the bird are summarized in Table 1, and the radar simulation parameters are listed in Table 2. The remaining experimental parameters for the rotary-wing UAV and the bird are provided in Table 3. The initial phase values of each blade were set randomly. Generally, the average power of the UAV fuselage is approximately 10 dB higher than that of the rotor; therefore, the power ratio between the UAV fuselage and the rotor sections was set to 10 dB. Similarly, for comparison purposes, the power ratio between the bird’s body and wings was also set to 10 dB. Since the signals from both the fuselage and the bird body are suppressed during processing, this value does not affect the SNR of the rotor and wing motion components.

#### 4.1.2. Experimental Platform and Parameters

All experiments were conducted on a workstation equipped with an Intel Core i7-12700F twelve-core CPU and an NVIDIA GeForce RTX 3060 Ti GPU, the manufacturer is Intel Corporation. The software environment included Python 3.9.13, CUDA 12.4, and PyTorch 1.13.1.

It should be noted that the proposed method is based on a weighted GNB classifier framework, which does not require deep learning-based training. However, to ensure consistency with comparison deep learning methods, the PyTorch framework was employed for data management and batch processing. The training parameters for the reference deep learning baselines were set as follows: 100 epochs, a batch size of 16, and the Adam optimizer with an initial learning rate of 0.01. Input spectrograms were normalized to 640 × 640 pixels when required by CNN-based comparison methods.

#### 4.1.3. Evaluation Metrics and Validation Strategy

Classification accuracy is used as the primary quantitative metric for UAV–bird classification. Confusion matrices are also employed under representative low-SNR conditions to examine class-specific recognition and misclassification patterns.

For each SNR level, the dataset generated in each Monte Carlo trial is randomly divided into ten subsets. A 10-fold cross-validation scheme is then applied. Each subset is used once as the test fold, while the remaining nine subsets are used for training. The training and test partition is determined before any data-dependent processing. Feature normalization, feature selection, feature-weight estimation, and weighted Gaussian Naive Bayes parameter estimation are performed using the current training fold only. The resulting parameters remain fixed when processing and classifying the corresponding test fold.

To reduce the influence of noise randomness and data partitioning, 50 independent Monte Carlo trials are conducted at each SNR level. Data generation, 10-fold partitioning, and cross-validation are performed independently in each trial. Samples from different Monte Carlo trials are not pooled for training or testing. For each trial, the classification accuracy is first averaged over the ten test folds. The final performance is then obtained by averaging the results over the 50 independent trials.

The random cross-validation strategy evaluates the overall performance under the current simulation setting. However, samples with similar physical parameter configurations may still appear in different folds. Therefore, the generalization capability to completely unseen motion configurations has not been fully evaluated in the present study. This issue can be further investigated using group-wise validation based on parameters such as rotor speed, wing beat frequency, target attitude, or motion state.

All compared methods use the same data partitions and training–test samples to maintain consistent evaluation conditions.

Classification accuracy is defined by Formula (33).

(33) $$\mathrm{Accuracy}=\frac{N_{\mathrm{correct}}}{N_{\mathrm{test}}}\times 100\%$$

where $N_{\mathrm{correct}}$ denotes the number of correctly classified test samples and $N_{\mathrm{test}}$ denotes the total number of test samples.

Based on this validation framework, the following sections analyze the classification performance of different features and methods under different SNR conditions.

### 4.2. Analysis of Classification Characteristics

Based on the two-scale feature extraction framework described in Section 3, six candidate feature families are constructed from the VMD modal domain and the STFT time–frequency subband domain. These include the modal energy ratio, frequency discrete entropy, singular value ratio, subband mean, subband standard deviation, and subband entropy. Following the validation strategy described in Section 4.1, candidate feature evaluation and selection are performed using the current training fold only.

This section analyzes the discriminative characteristics of the candidate features for UAV–bird classification. First, the classification performance of the three VMD-based feature families is compared. The three STFT-based statistical feature families are then evaluated. Different cross-domain feature combinations are further examined to assess the complementary information provided by the VMD and STFT representations. Based on these analyses, the modal energy ratio and STFT subband mean are retained for subsequent classification.

The following analysis focuses on feature distributions, classification accuracy, and the performance of different feature combinations under different SNR conditions.

According to the radar parameters listed in Table 2, the pulse repetition interval is 50 us, corresponding to a nominal pulse repetition frequency of 20 kHz. For clearer visualization of the target micro-Doppler characteristics, Figure 10 and Figure 11 display only the frequency region containing the dominant micro-Doppler components. Therefore, the frequency axes in these figures represent the micro-Doppler region of interest rather than the full frequency range associated with the radar PRF.

Figure 10 presents the time–frequency spectra and VMD modal energy distributions under noise-free conditions. UAV rotor signals exhibit clear harmonic structures in the time–frequency domain, with energy distributed across multiple VMD modes. In comparison, bird wing-flapping signals show more concentrated low-frequency patterns, with most of the modal energy distributed in a smaller number of low-order modes. These differences reflect the distinct micro-motion characteristics of UAV rotors and bird wings and provide useful information for feature extraction. As the SNR decreases, the corresponding time–frequency and modal structures become less distinct due to noise interference. This observation motivates the use of complementary feature representations from both the VMD modal domain and the STFT time–frequency domain.

Figure 11 presents the time–frequency spectra and VMD modal energy distributions of UAV and bird signals at SNR levels of 10 dB, 0 dB, and −5 dB. The displayed frequency axes correspond to the micro-Doppler region of interest defined above. At high SNR, UAV signals show clear harmonic structures, while bird signals exhibit more concentrated low-frequency patterns. As the SNR decreases, these structures become increasingly obscured by noise, which reduces their separability in the time–frequency domain.

The VMD modal energy distributions show relatively stable characteristics across the evaluated SNR levels. UAV signals generally exhibit energy distributed over multiple modes, while bird signals tend to show a more concentrated energy distribution in a smaller number of modes. Although some modal spreading appears as the noise level increases, the difference in modal energy distribution remains observable under low-SNR conditions. This provides useful statistical information for target discrimination. The STFT subband features provide complementary information from the time–frequency domain. By dividing the spectrum into frequency subbands and calculating statistical descriptors, local variations in micro-Doppler energy can be characterized. UAV and bird signals show different energy distributions across the frequency subbands because of their different micro-motion mechanisms. These differences provide additional information for the subsequent classification stage. Figure 12 further shows the distributions of the subband statistical features for UAV rotor signals, bird wing-flapping signals, and Gaussian white noise under noise-free conditions. Only the micro-motion components are considered in this analysis. Platform-body echoes and ground clutter are not included.

As shown in Figure 12, both UAV and bird signals exhibit strong energy concentration in the first subband. The mean value of the UAV rotor signal in this subband is close to one, while that of the bird wing-flapping signal is lower, at approximately 0.8. From the second subband onward, both the mean and standard deviation decrease rapidly, indicating that most of the micro-motion energy is concentrated in the low-frequency region. In comparison, Gaussian white noise shows a more uniform distribution across the frequency subbands, without a clear dominant subband. The subband entropy of the noise remains relatively high over most frequency regions. For the UAV and bird signals, lower entropy values are observed in the low-frequency subbands, while the entropy tends to increase toward higher-frequency regions. This trend indicates that the low-frequency components contain more structured micro-motion information, whereas the higher-frequency components are increasingly affected by noise. Overall, the UAV rotor signal shows a broader low-frequency energy distribution than the bird wing-flapping signal, while both exhibit different subband statistics from Gaussian white noise. These observations indicate that the subband mean, standard deviation, and entropy provide useful statistical descriptions of the micro-motion signals. Their variations under different SNR conditions are further analyzed in Figure 13 and Figure 14.

As the SNR decreases, the differences among the subband statistical features become less pronounced because the micro-Doppler components are increasingly affected by noise. However, different distribution patterns can still be observed for UAV and bird signals. For UAV rotor signals, the energy is distributed over several low-frequency subbands, with relatively large mean values in the first few subbands. As the SNR decreases, the mean and standard deviation of these subbands decrease, but the energy distribution remains relatively spread across multiple low-frequency regions.

In comparison, bird wing-flapping signals show a more concentrated energy distribution, with the first subband generally containing a larger proportion of the micro-motion energy. The remaining subbands exhibit smaller statistical variations. The entropy values of both target types become more similar as the SNR decreases, indicating that noise reduces the structural differences in the time–frequency representation. Nevertheless, the UAV signals tend to maintain a broader subband energy distribution, while the bird signals remain more concentrated in fewer low-frequency subbands. These observations are consistent with the modal energy characteristics discussed in the VMD analysis and indicate that the subband statistical features retain useful discriminative information under the evaluated low-SNR conditions. To further quantify the contribution of the candidate features, their classification performance is evaluated using the validation framework described in Section 4.1.3. The VMD-based candidate feature families and the STFT-based candidate feature families are first evaluated separately. Different feature combinations are then compared to examine their complementary information and their contribution to UAV–bird classification. All feature evaluation and selection procedures are performed using the training fold only.

The above analysis shows that the extracted features exhibit different statistical patterns for UAV and bird signals. The following section provides a quantitative comparison of the candidate features and their combinations under different SNR conditions.

### 4.3. Classification Experiment Results and Analysis

#### 4.3.1. Candidate Feature Evaluation

The classification performance of the candidate feature families is evaluated under different SNR conditions. For the VMD branch, the modal energy ratio (MER), frequency discrete entropy (FDE), singular value ratio (SVR), and their joint representation are compared. For the STFT branch, the subband mean, subband standard deviation, subband entropy, and their joint representation are evaluated in the same manner. As shown in Table 4, the MER achieves the best overall performance among the three individual VMD-based feature families. Its classification accuracy is higher than that of FDE and SVR at most SNR levels. The joint VMD feature set provides further improvement, especially as the SNR decreases. These results indicate that MER contains the most useful discriminative information among the individual VMD-based candidates. As shown in Table 5, the subband mean achieves the best overall performance among the three individual STFT-based feature families. It provides the highest average accuracy over the evaluated SNR range. The subband standard deviation performs slightly better at some extremely low-SNR conditions, while the subband mean shows more consistent overall performance. The joint STFT feature set provides a further improvement over the individual features.

Based on the above comparison, MER is selected as the representative VMD-based feature family, while the subband mean is selected as the representative STFT-based feature family. MER describes the distribution of micro-motion energy across VMD modes, whereas the subband mean describes the average micro-Doppler energy across STFT frequency subbands. The two feature families therefore provide complementary information from different representation domains. Considering classification performance and feature redundancy, the selected MER and subband mean features are used to construct the final fused feature vector for subsequent classification. This selection retains useful discriminative information while avoiding unnecessary candidate feature inputs.

#### 4.3.2. Ablation Experiment Analysis

To further evaluate the contribution of the selected feature families, different feature configurations are compared under the experimental framework described in Section 4.1. The comparison includes all six candidate feature families, the VMD modal energy ratio (MER), the STFT subband mean, and the final model based on the selected MER and subband mean features with weighted Gaussian Naive Bayes.

As shown in Table 6, the full candidate feature set achieves the best overall classification performance, but it also contains all six candidate feature families and therefore retains more redundant information. After feature selection, MER and subband mean are retained as the representative VMD-based and STFT-based feature families. The final model maintains competitive performance across the evaluated SNR range while using a more compact cross-domain feature representation.

As shown in Figure 15, the classification performance of all VMD-based feature families decreases as the SNR decreases. Among the individual VMD features, MER shows the best overall performance across the evaluated SNR range. The joint VMD feature set performs better than the individual features, indicating that the three VMD-based feature families contain partially complementary information. A similar trend is observed for the STFT-based features in Figure 16. The subband mean provides the best overall performance among the individual STFT feature families, while the subband standard deviation performs slightly better under some extremely low-SNR conditions. The joint STFT feature set generally provides better performance than the individual features, suggesting that the statistical descriptors capture complementary aspects of the time–frequency energy distribution.

Overall, the candidate feature families contribute different levels of discriminative information. MER and subband mean show the strongest overall performance among the individual VMD-based and STFT-based features, respectively. Their combination forms a compact cross-domain representation for the final classifier. Although the full candidate feature set can achieve slightly higher accuracy under some SNR conditions, the selected feature configuration reduces redundant inputs while preserving competitive classification performance.

#### 4.3.3. Comparison of Classification Performance Under Low Signal-to-Noise Ratio

Due to the significant difference in the maximum Doppler frequency range between the rotor motion of UAVs and the wing-flapping motion of birds, the spectral width can be intuitively used to distinguish between these two types of target. However, under low-SNR conditions, this method is highly susceptible to noise interference, which may lead to distortions in spectral width estimation and consequently degrade classification reliability. To evaluate this limitation, comparative experiments were conducted based on the dataset and platform configuration described in Section 4.1. In accordance with the SNR definition in Section 3.1, the following experiments use $SNR_{m}$ as the unified SNR measure. Particular attention is paid to $SNR_{m}\leq -10$ dB, where the micro-motion components are severely masked by noise.

Figure 17 shows the estimated micro-Doppler spectral widths of UAV and bird echoes under different SNR conditions. As the SNR decreases, the estimated spectral width becomes increasingly affected by noise and may expand toward the boundary of the Doppler range. This behavior indicates that the original micro-motion structure becomes less distinguishable under severe noise interference, which reduces the reliability of spectral-width-based characterization. The classification performance of the proposed method and several baseline approaches under different SNR conditions is compared in Figure 18. As shown in Figure 18, the classification accuracy of all methods decreases as the SNR decreases. The proposed method achieves the best overall performance among the evaluated methods and shows a clearer advantage under low-SNR conditions. In particular, it still achieves an accuracy of 80.2% at −20 dB. In comparison, the CNN-based, conventional STFT-based, and raw-feature methods exhibit greater performance degradation as the noise level increases. At higher SNR levels, the performance differences among the methods become smaller. Overall, the results indicate that the proposed VMD-STFT framework retains useful discriminative information under low-SNR conditions and provides competitive classification performance across the evaluated SNR range.

Wavelet-based features have been used for radar target classification [29]. EMD has also been applied to micro-Doppler signal extraction for small UAV detection [30]. Based on these studies, DWT-SVD and EMD are selected as comparison methods in this study. All methods are evaluated under the same weighted GNB classifier framework and use the same 10-fold cross-validation partitions and Monte Carlo trials described in Section 4.1. The reported results are averaged over all trials. As shown in Figure 19, the classification accuracy of all methods decreases as the SNR decreases. The DWT–SVD and EMD methods show a more pronounced performance decline in the low-SNR region. In comparison, the proposed method exhibits a more gradual decrease in classification accuracy and maintains better overall performance under the evaluated low-SNR conditions.

The comparison further indicates that the VMD-based and STFT-based features provide complementary descriptions of target micro-motion characteristics. The VMD features characterize the modal energy distribution, while the STFT features describe the energy distribution in different time–frequency subbands. Their combination provides complementary information for subsequent weighted GNB classifier.

Figure 20 compares the class-wise classification results obtained using the VMD-based features, STFT-based features, and the fused VMD–STFT representation under the low-SNR condition of −5 dB. The two single-domain feature representations exhibit more cross-class misclassifications between bird and rotor-UAV samples. In comparison, the fused feature representation shows a clearer concentration along the main diagonal and fewer misclassified samples. This result indicates that the VMD and STFT features provide complementary information for distinguishing the two target classes under low-SNR conditions.

To further examine the discriminative characteristics of the extracted features, the feature distributions of different methods at SNR = 10 dB and SNR = −5 dB are visualized in Figure 21.

As shown in Figure 21, the VMD-based and STFT-based single-domain features exhibit different degrees of overlap between UAV and bird samples under low-SNR conditions. The class boundaries become less distinct as the extracted features are increasingly affected by noise. In comparison, the fused VMD–STFT representation shows clearer separation between the two target classes. Although the sample distributions become more dispersed under noise interference, the UAV and bird samples remain distinguishable in the fused feature space. These results indicate that the VMD and STFT feature representations contain complementary information. Their combination integrates modal energy characteristics with time–frequency subband information and provides a more informative representation for UAV–bird classification under low-SNR conditions.

In summary, the proposed method integrates VMD-based modal features and STFT-based subband statistical features within a weighted Gaussian Naive Bayes classification framework. The two feature domains describe different aspects of target micro-motion characteristics and provide complementary information for classification. The resulting fused representation supports effective discrimination between rotary-wing UAVs and birds and maintains competitive classification performance under the evaluated low-SNR conditions.

## 5. Conclusions

This study investigates the challenging problem of discriminating between rotary-wing UAVs and birds in low-altitude radar surveillance under low-SNR conditions. By exploiting the differences between UAV rotor modulation and bird wing-flapping motion in terms of micro-Doppler characteristics and modal energy distributions, a weighted GNB classifier framework based on the fusion of VMD-derived modal features and STFT-based time–frequency subband statistical features is developed. Through candidate feature construction and feature selection, the VMD modal energy ratio and STFT subband mean are retained to form a compact cross-domain feature representation, which is subsequently classified using a weighted Gaussian Naive Bayes model. The main findings of this study can be summarized as follows:

(1) The proposed feature-selection and fusion strategy maintain competitive classification performance under low-SNR conditions while reducing redundant feature inputs. The experimental results show that the proposed method achieves a classification accuracy of approximately 80.2% at an SNR of −20 dB, with performance improving steadily as the SNR increases. Compared with directly using all candidate feature families, the selected feature representation provides a more compact input space while preserving effective discriminative information.

(2) The VMD modal energy ratio and STFT subband mean capture complementary characteristics of target micro-motion from the modal decomposition and time–frequency analysis perspectives, respectively. By integrating these two feature domains, the proposed representation retains essential motion-related information while eliminating redundant feature components, providing a compact and effective representation for UAV–bird discrimination in low-SNR environments.

(3) The weighted Gaussian Naive Bayes classifier provides a unified probabilistic framework for modeling the selected multi-domain features. By incorporating differentiated feature contributions into the class-conditional probability model and estimating the corresponding Gaussian distributions, the classifier performs target discrimination according to the maximum a posteriori criterion. This formulation enables a concise and interpretable probabilistic decision process for the selected feature representation.

(4) The use of simulated radar echoes is an important limitation of the present study. In practical radar measurements, additional effects such as radar cross-section fluctuations, multipath propagation, non-stationary clutter, target attitude variations, and hardware imperfections may further influence the observed micro-Doppler signatures. Future work will therefore focus on validating the proposed method using measured UAV and bird radar data, as well as investigating adaptive feature extraction, heterogeneous information fusion, and multi-sensor cooperative perception in more complex low-altitude surveillance environments.

## Figures and Tables

**Figure 1:**
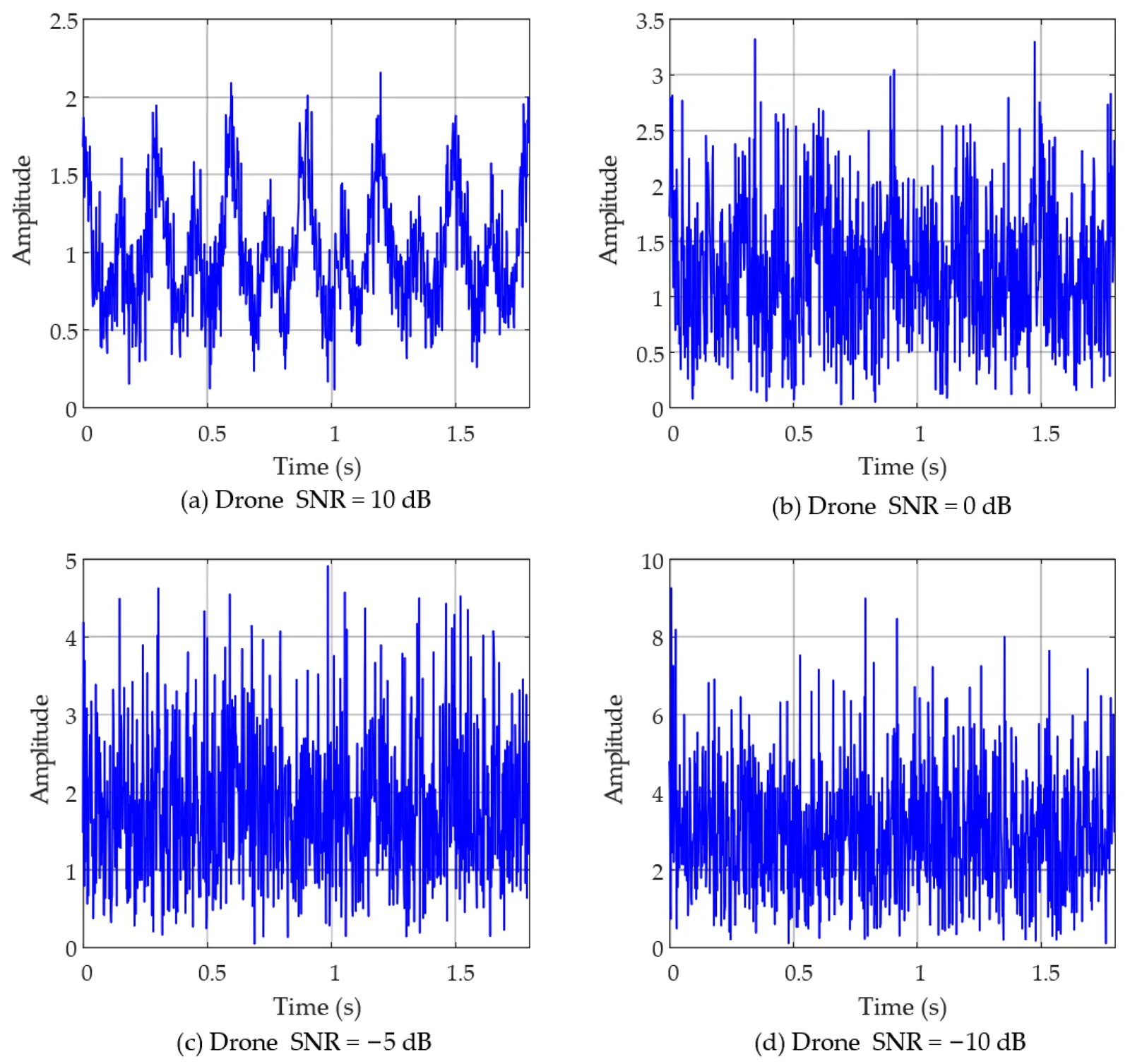
Time-domain waveforms of quadcopter radar echo signals under different SNR conditions.

**Figure 2:**
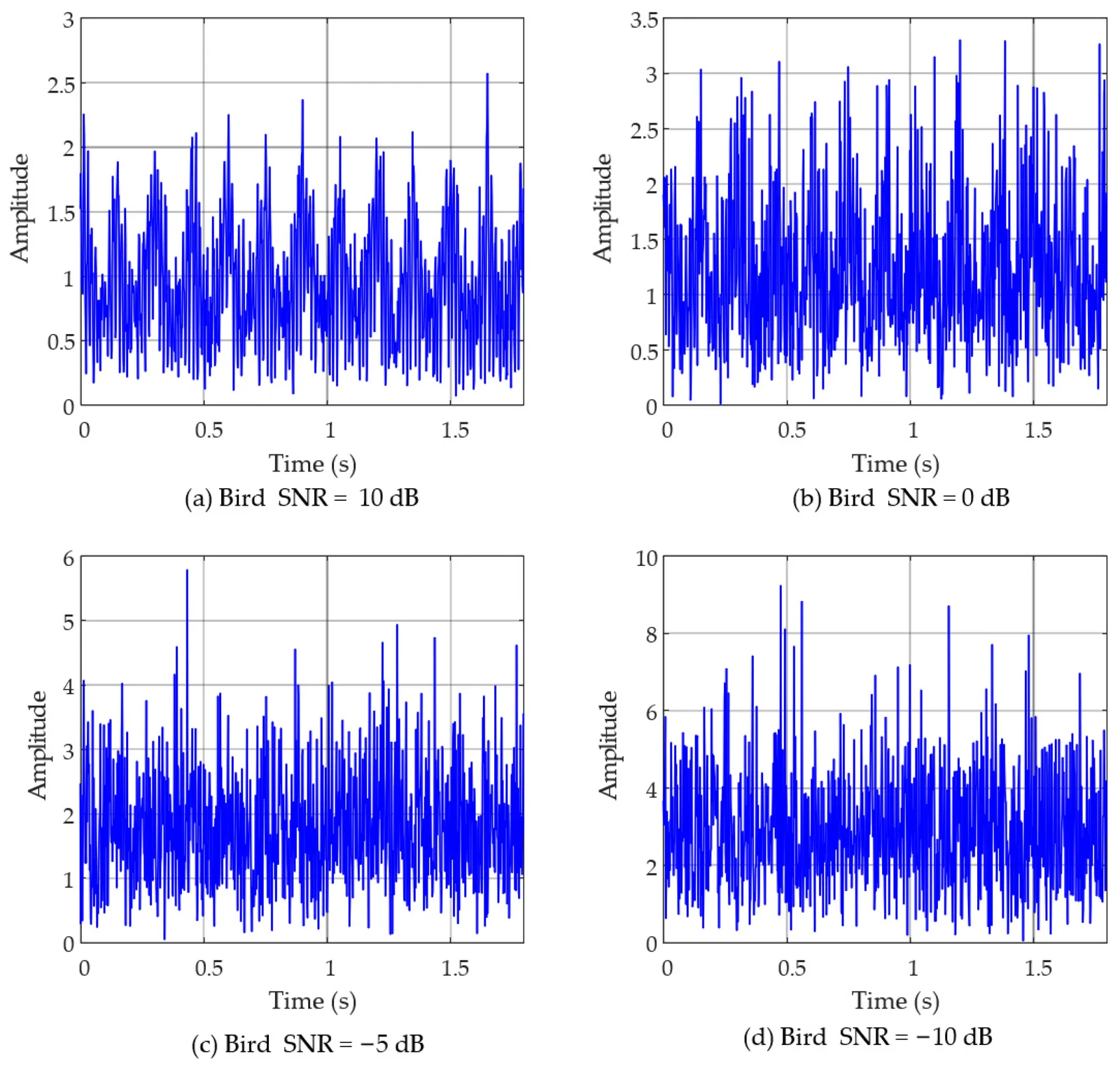
Time-domain waveforms of bird wing-flapping radar echo signals under different SNR conditions.

**Figure 3:**
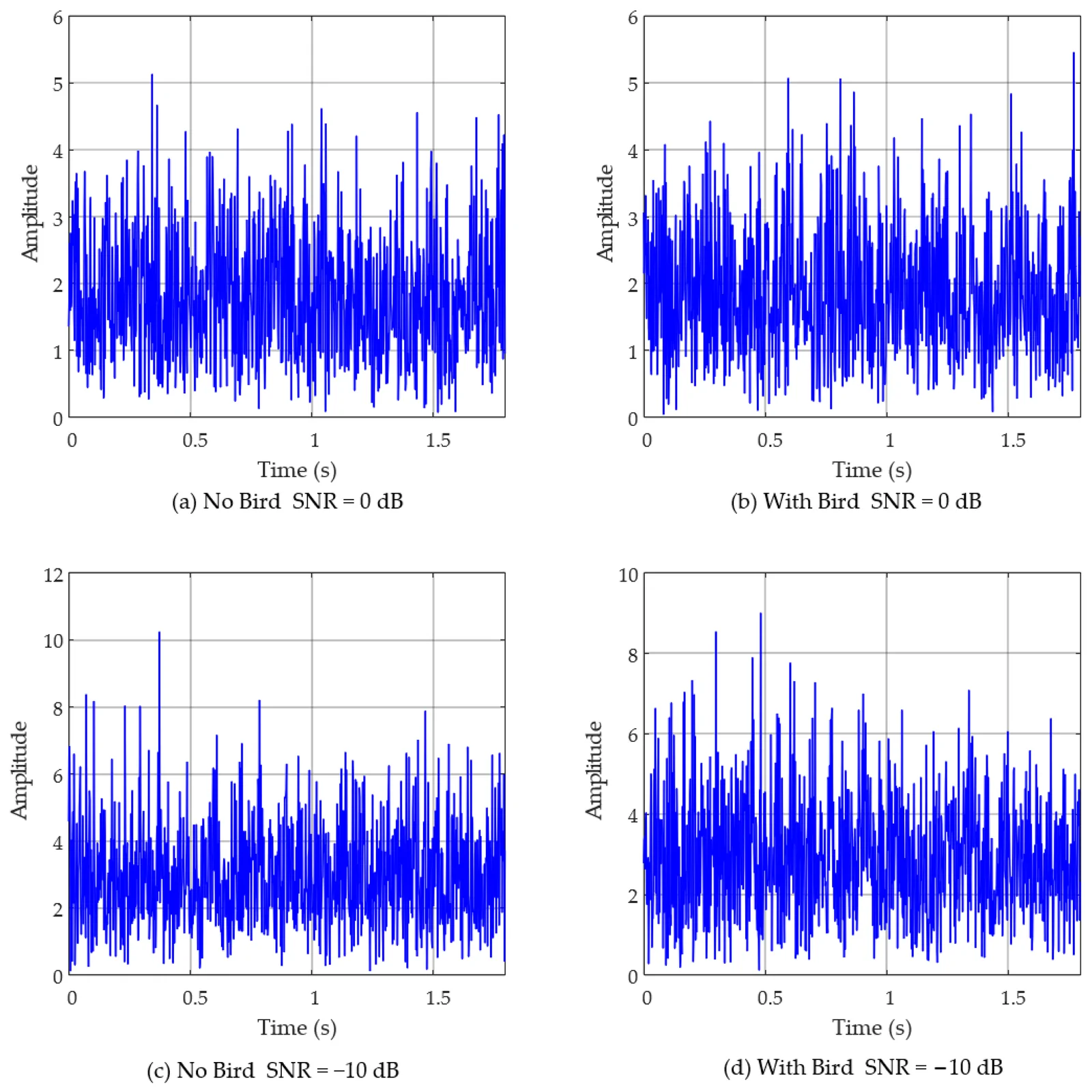
Time-domain waveform comparison matrix under different SNR conditions.

**Figure 4:**
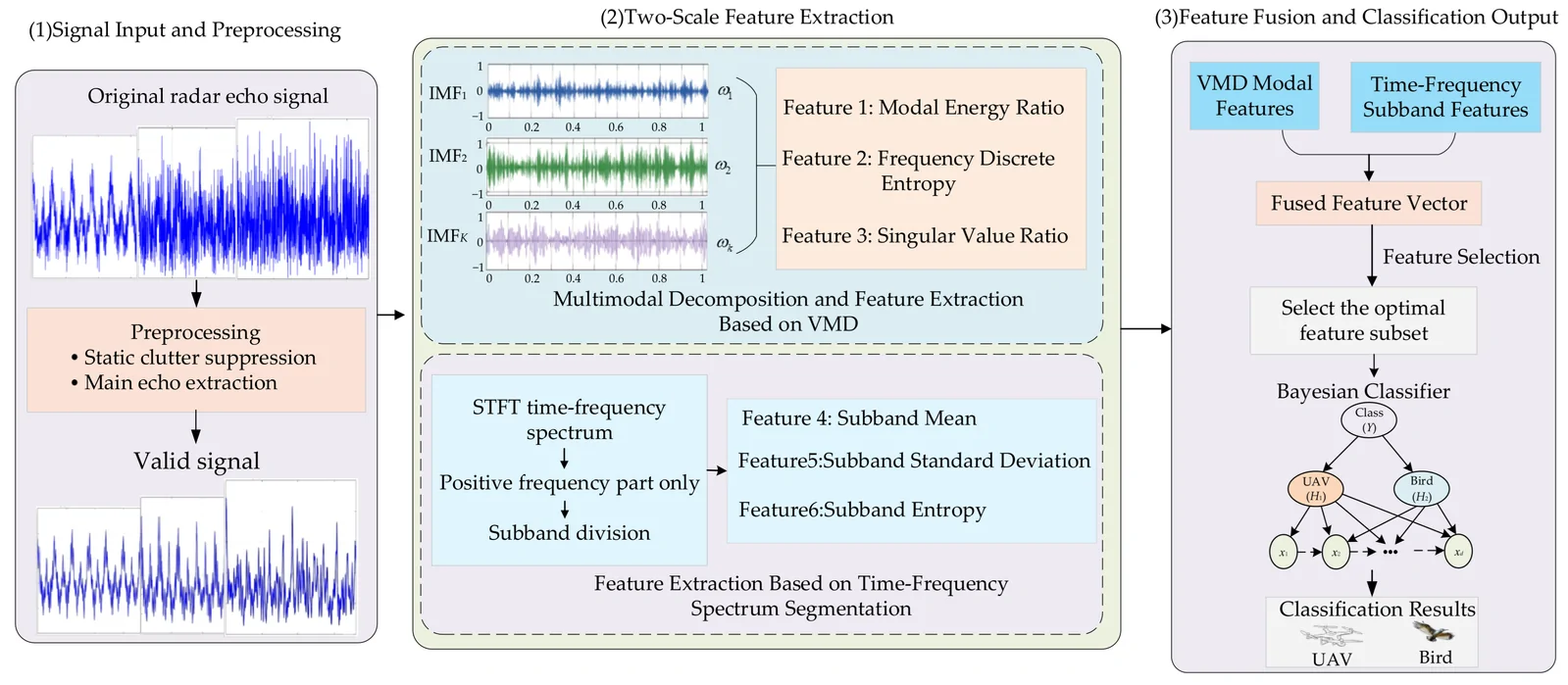
Overall Research Framework.

**Figure 5:**
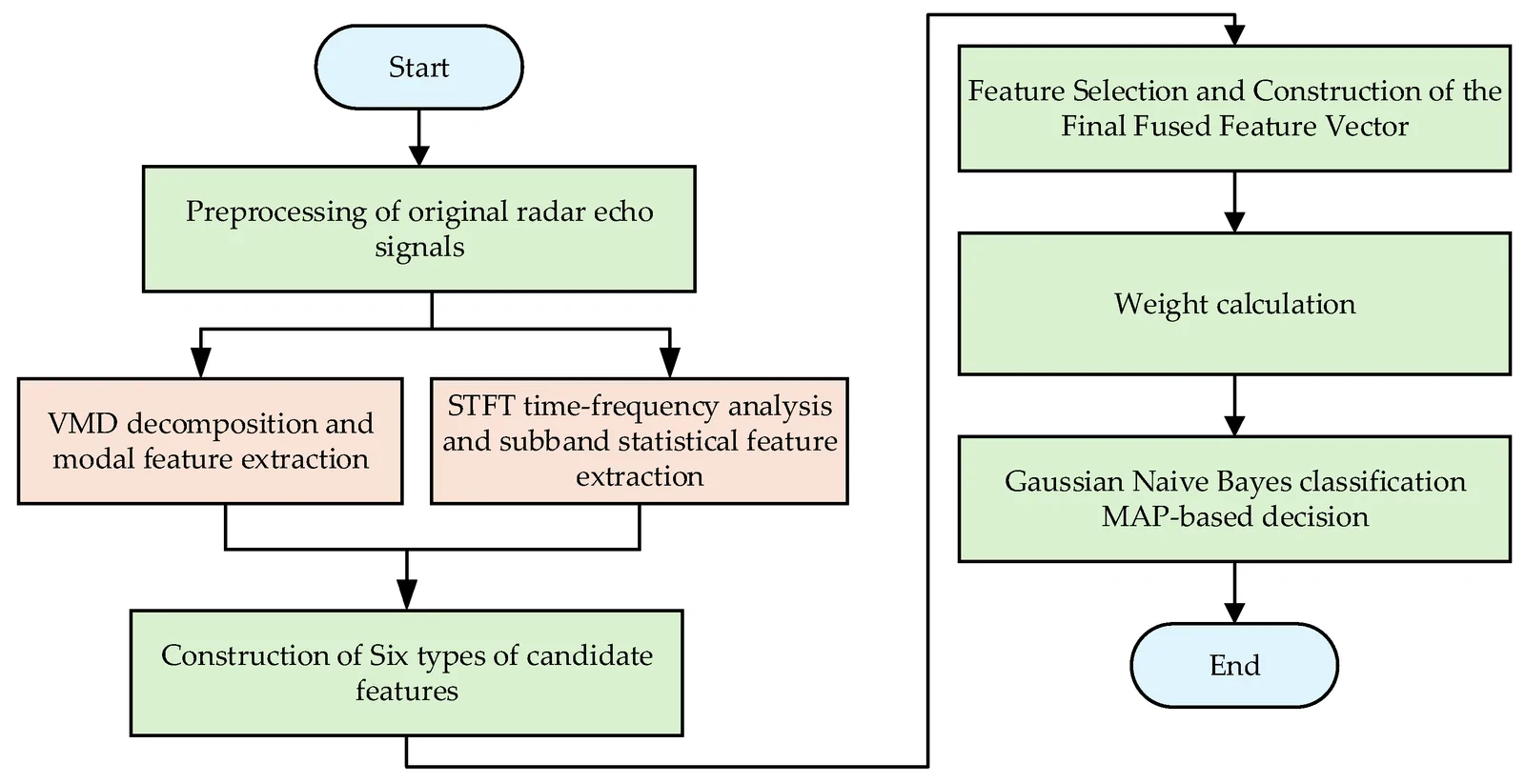
Overall flowchart of the proposed target recognition method based on VMD and time–frequency feature fusion.

**Figure 6:**
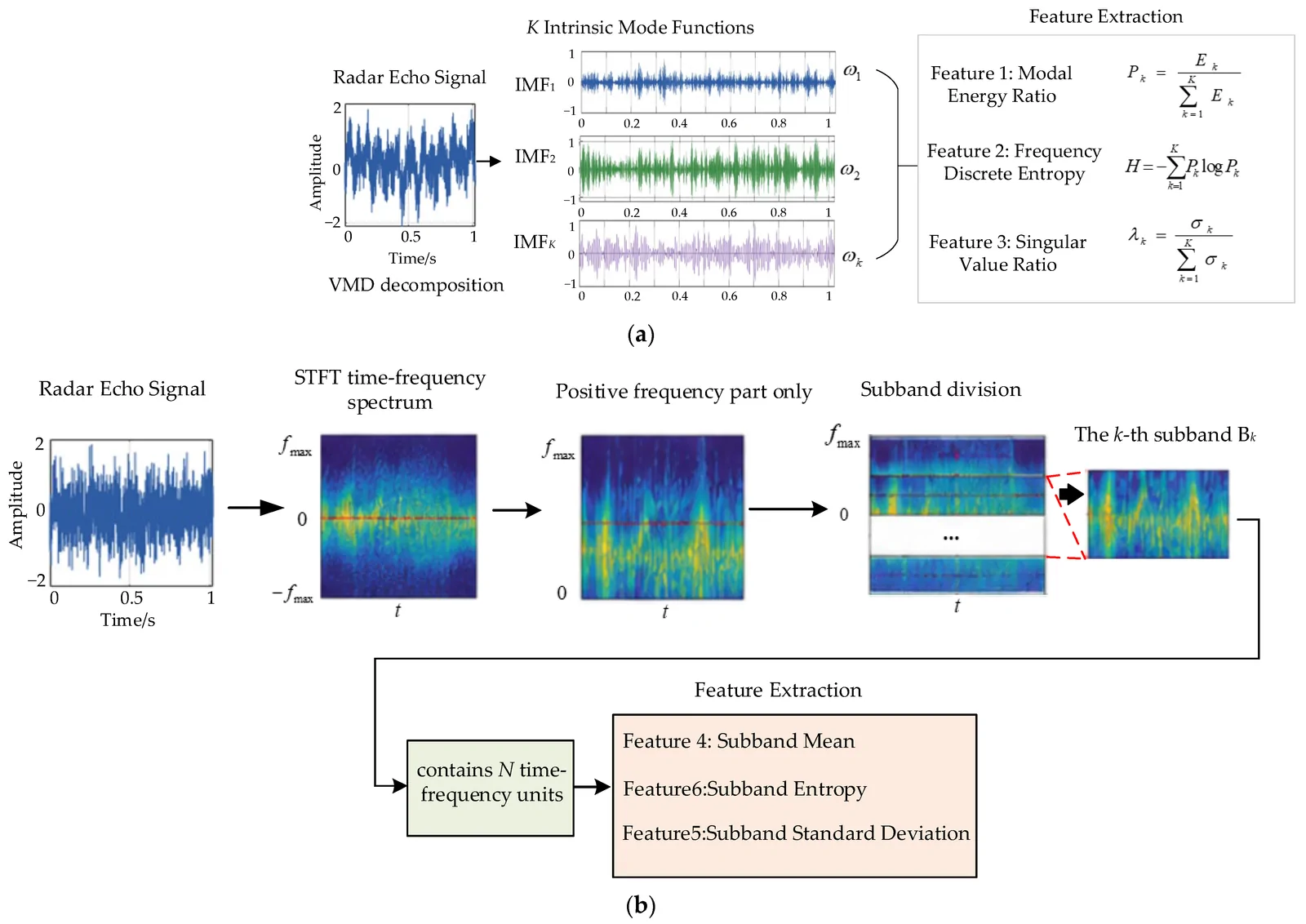
Two-scale feature extraction framework based on VMD and STFT. (**a**) VMD modal feature extraction. (**b**) STFT Time–Frequency Subband Statistical Feature Extraction.

**Figure 7:**
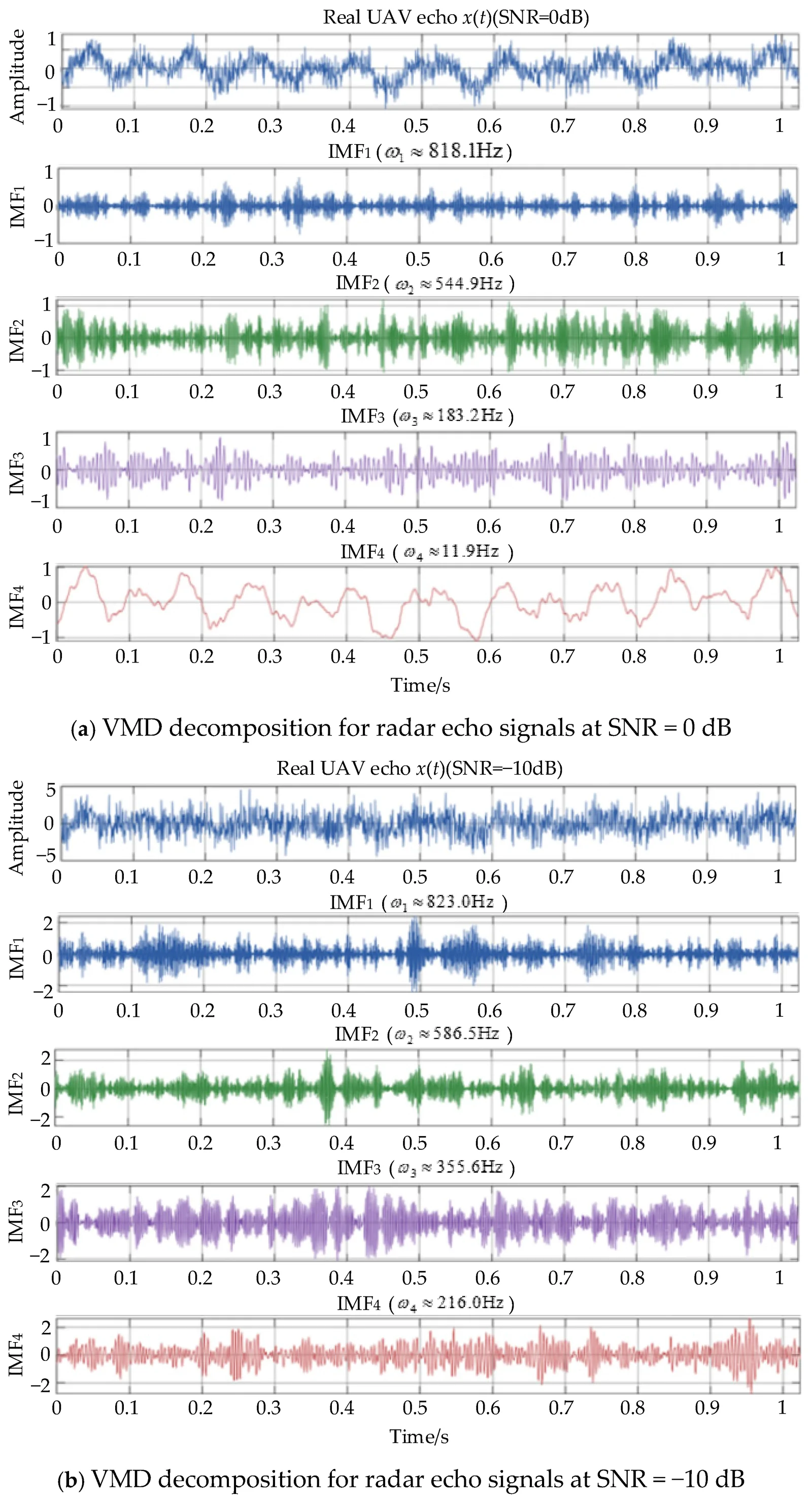
Example of VMD decomposition for radar echo signals at SNR = 0 dB and SNR = −10 dB.

**Figure 8:**
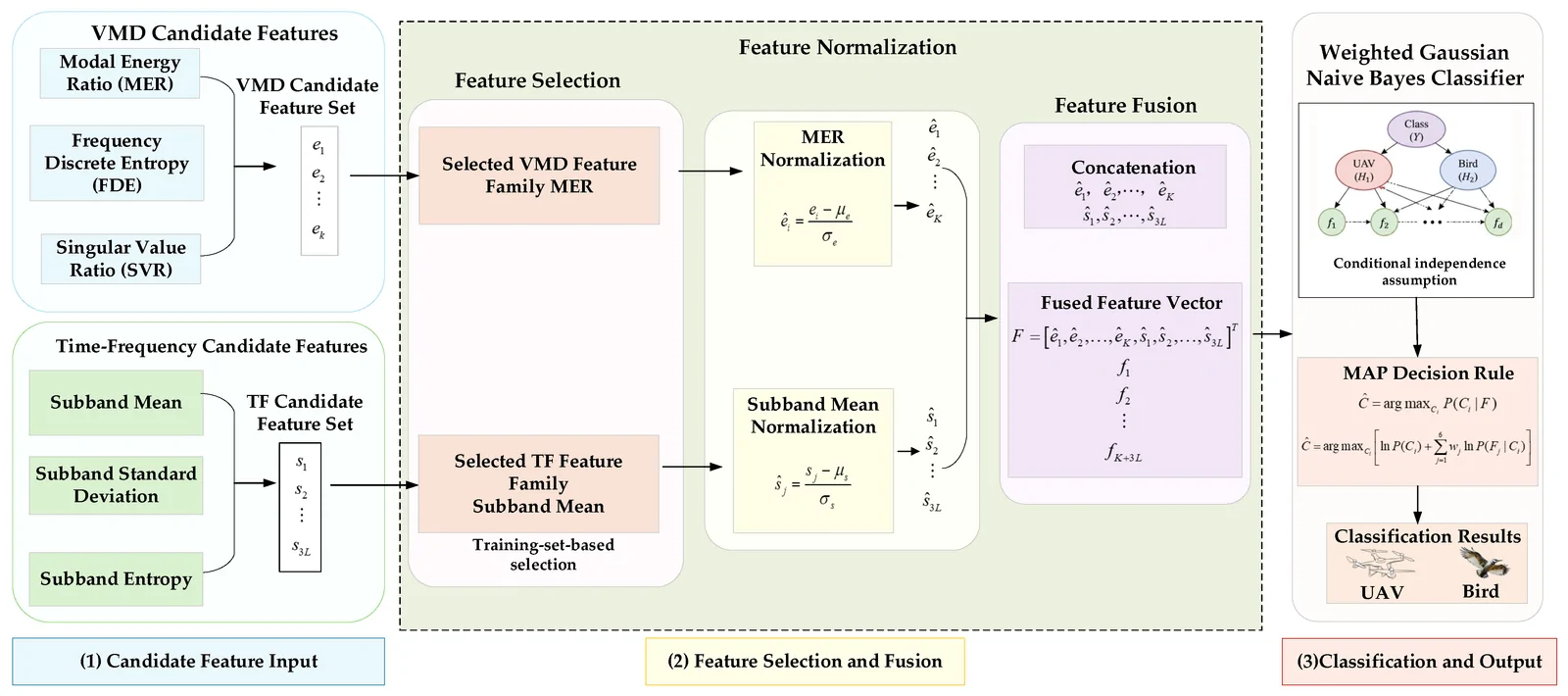
Framework of the proposed feature selection, fusion, and weighted GNB classifier method.

**Figure 9:**
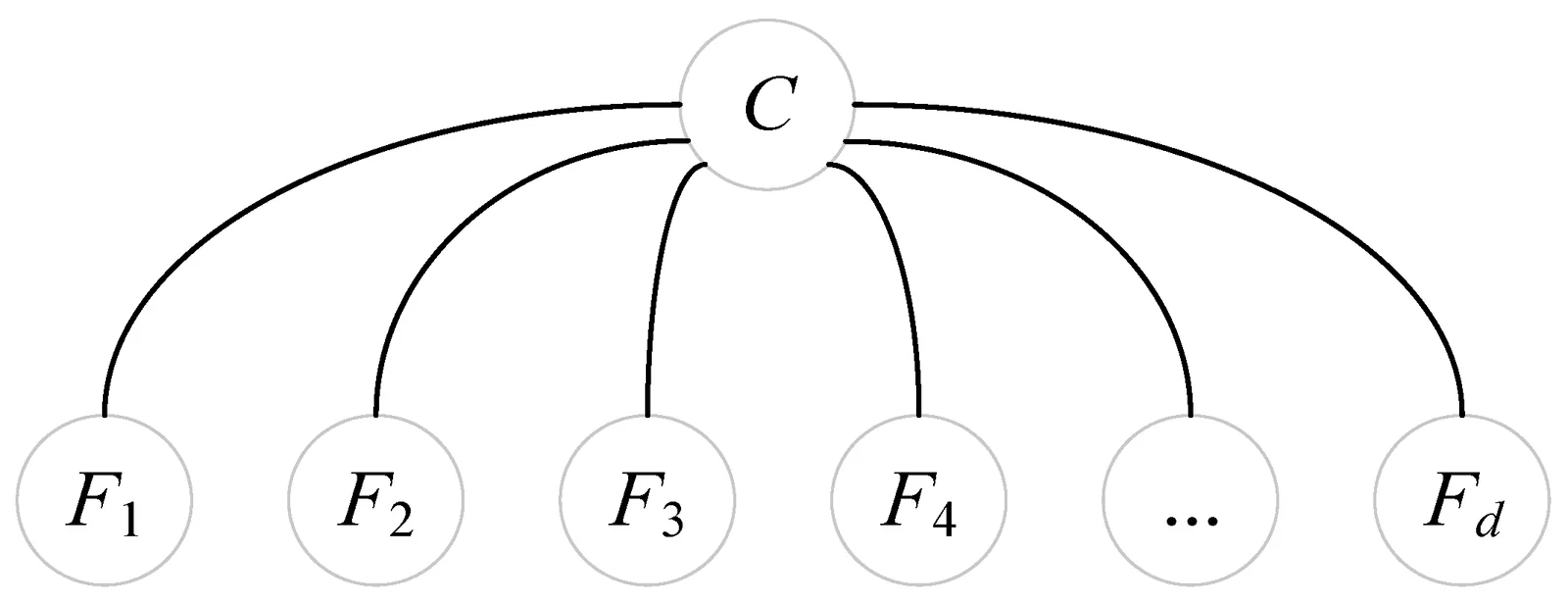
Topological relationships of the Naive Bayes network.

**Figure 10:**
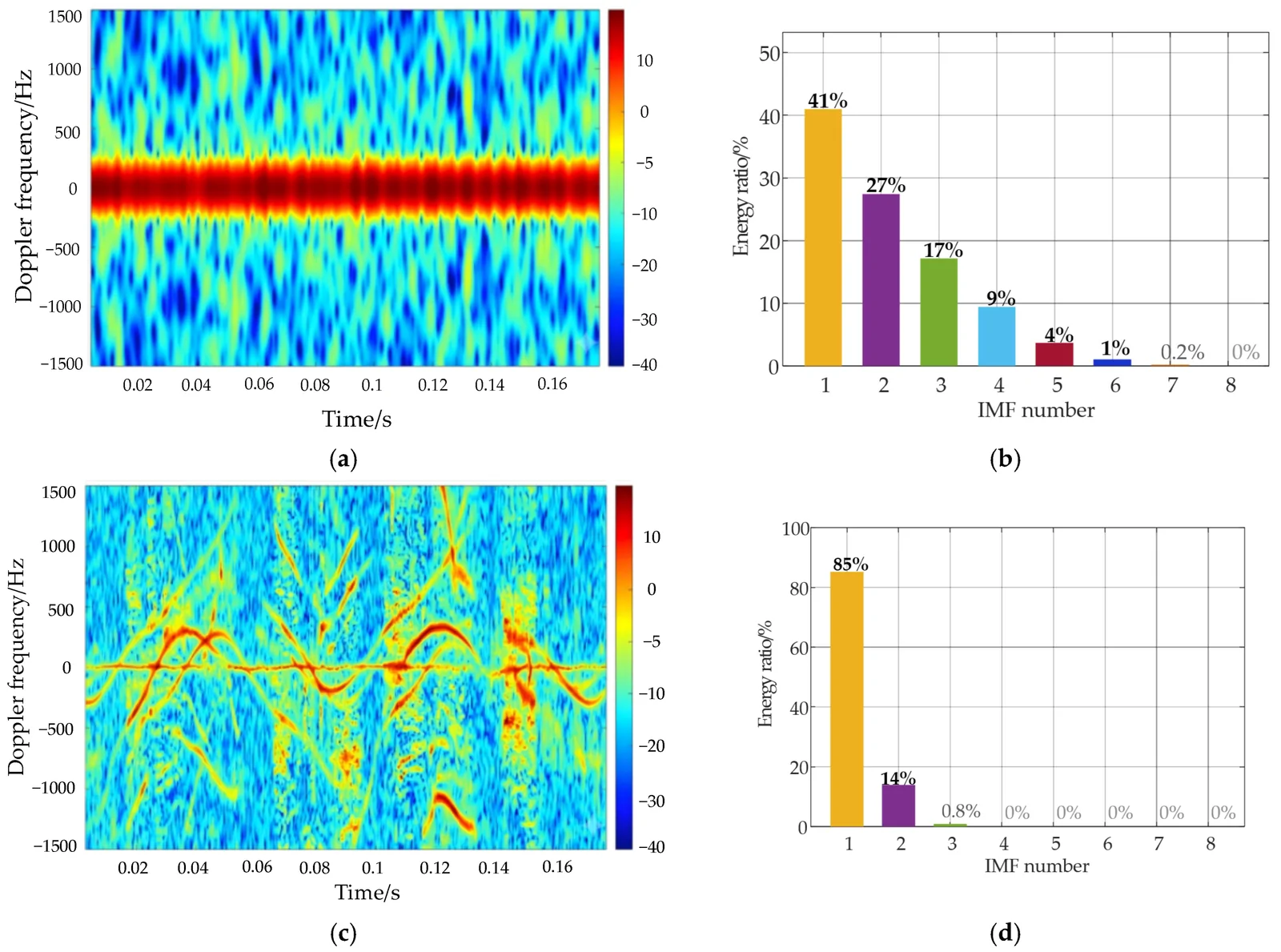
Time–frequency spectra and VMD modal energy distributions under noise-free conditions. (**a**) UAV rotor signal time–frequency spectrum. (**b**) Distribution of VMD mode energy ratio (%) for UAV rotor. (**c**) Bird flapping signal time–frequency spectrum. (**d**) Distribution of VMD mode energy ratio (%) for bird flapping.

**Figure 11:**
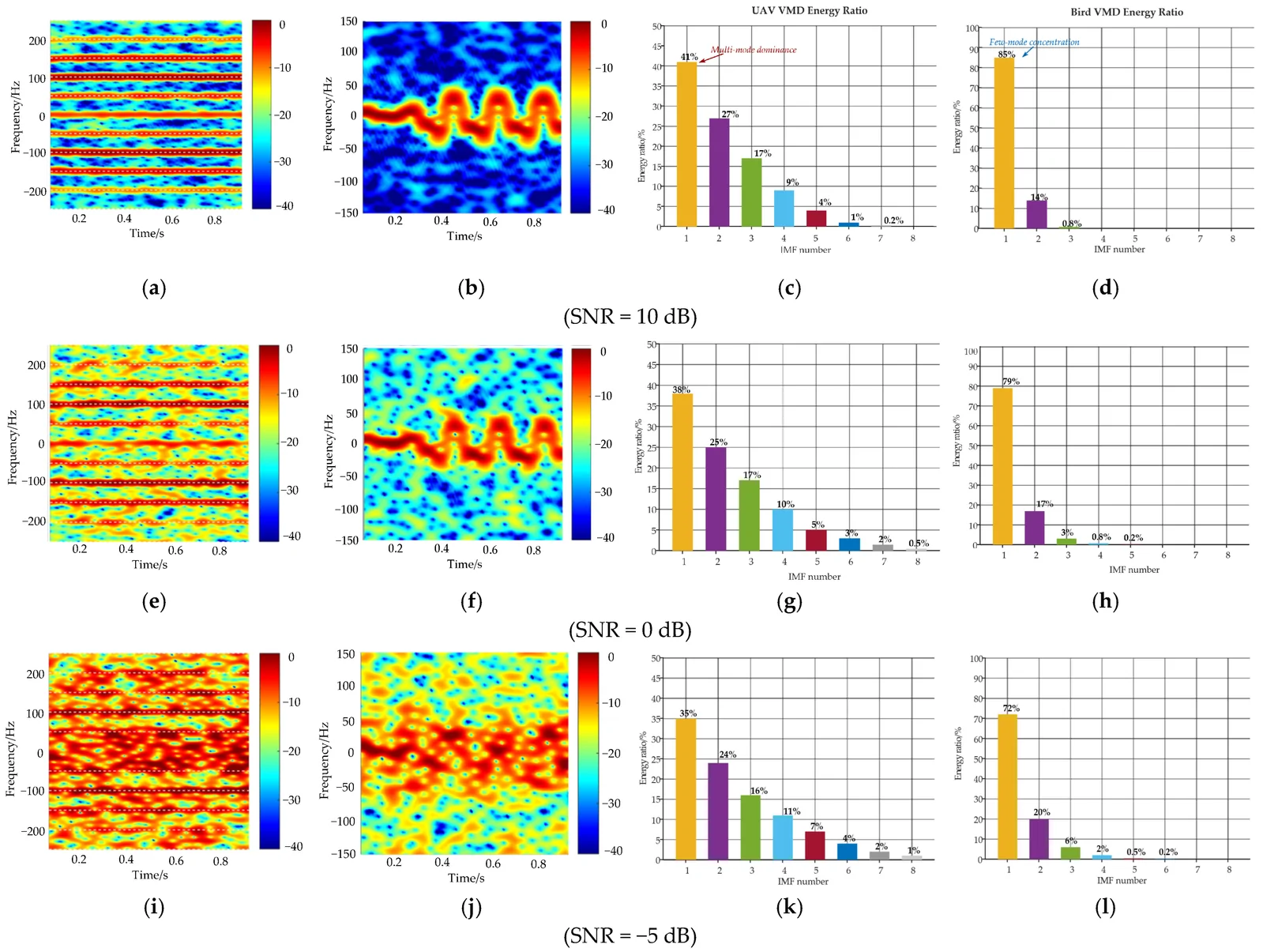
Time–frequency spectra and VMD modal energy distributions under different SNR conditions. (**a**) UAV Rotor TF Spectrum. (**b**) Bird Flapping TF Spectrum. (**c**) VMD Energy Ratio UAV. (**d**) VMD Energy Ratio Bird. (**e**) UAV Rotor TF Spectrum. (**f**) Bird Flapping TF Spectrum. (**g**) VMD Energy Ratio UAV. (**h**) VMD Energy Ratio Bird. (**i**) UAV Rotor TF Spectrum. (**j**) Bird Flapping TF Spectrum. (**k**) VMD Energy Ratio UAV. (**l**) VMD Energy Ratio Bird.

**Figure 12:**
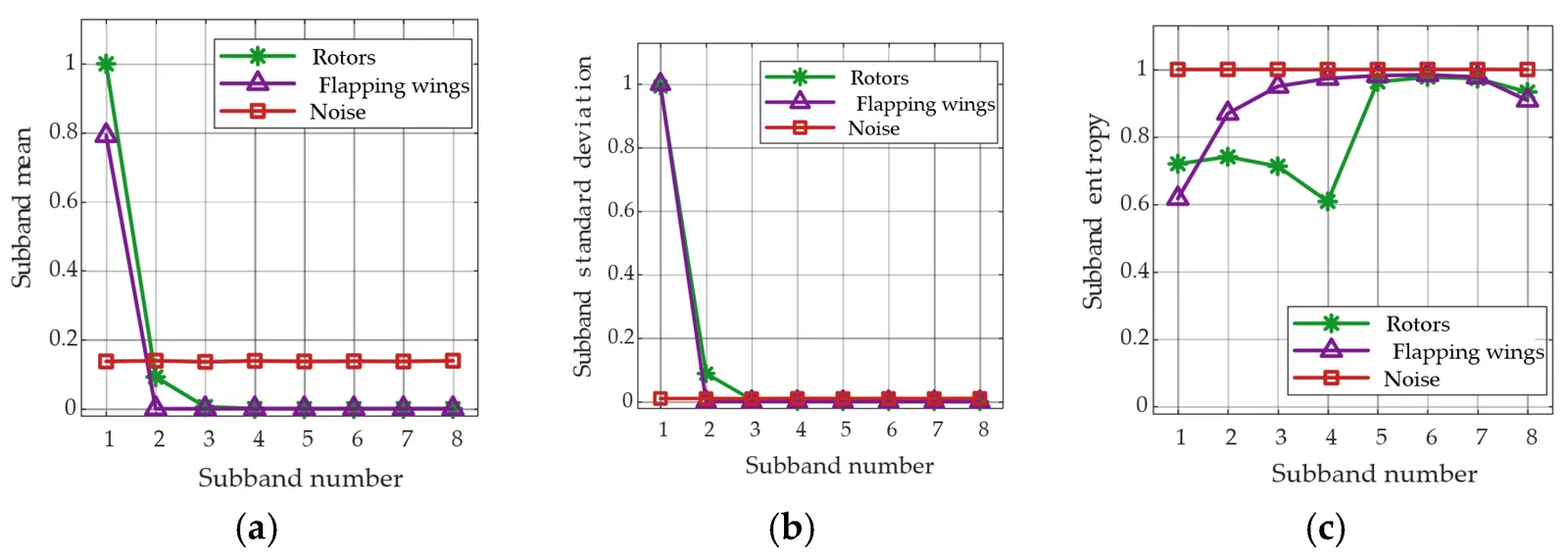
Statistical characteristics of the time–frequency subbands for UAV rotor signals, bird wing-flapping signals, and Gaussian white noise. (**a**) Subband mean. (**b**) Subband standard deviation. (**c**) Subband entropy.

**Figure 13:**
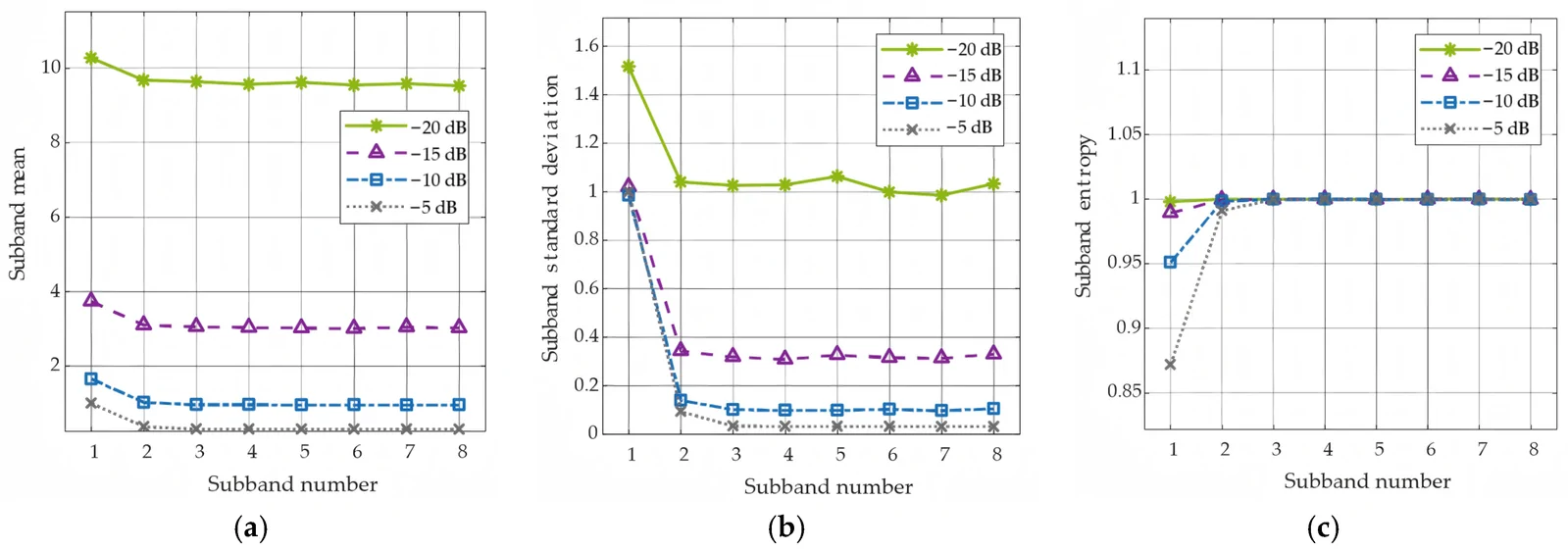
Statistical characteristics of the time–frequency subbands at different SNR levels for UAV rotor signals. (**a**) Subband mean. (**b**) Subband standard deviation. (**c**) Subband entropy.

**Figure 14:**
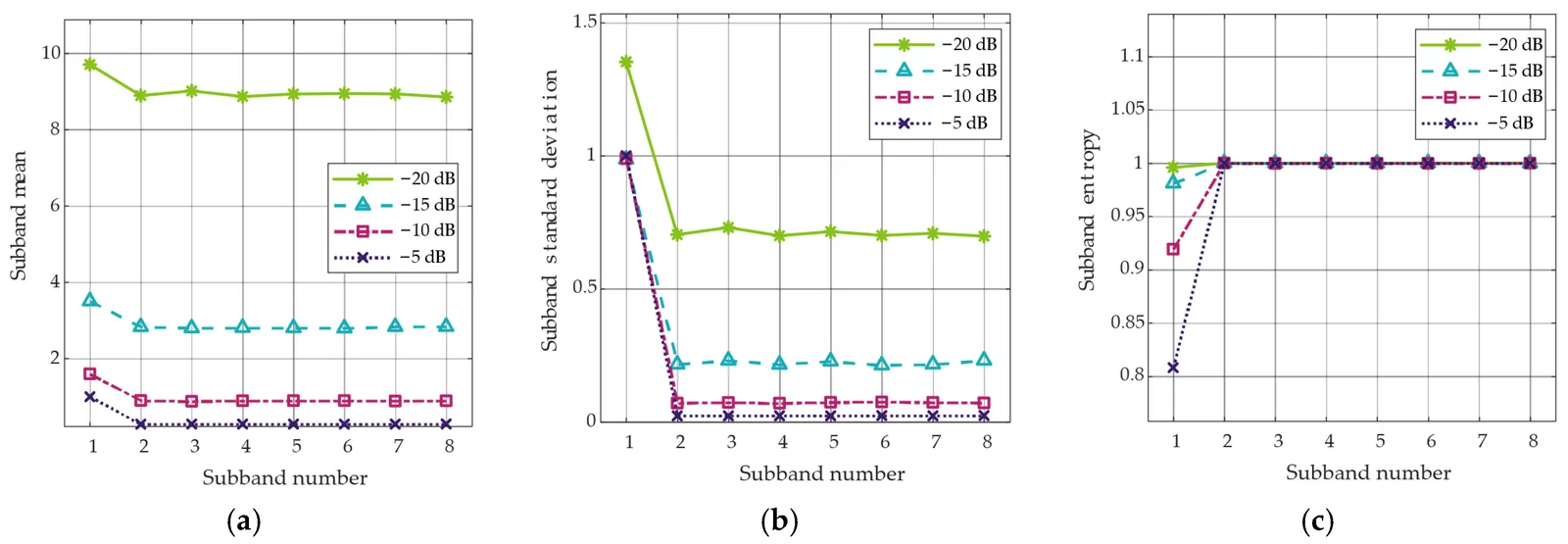
Statistical characteristics of the time–frequency subbands at different SNR levels for bird wing-flapping signals. (**a**) Subband mean. (**b**) Subband standard deviation. (**c**) Subband entropy.

**Figure 15:**
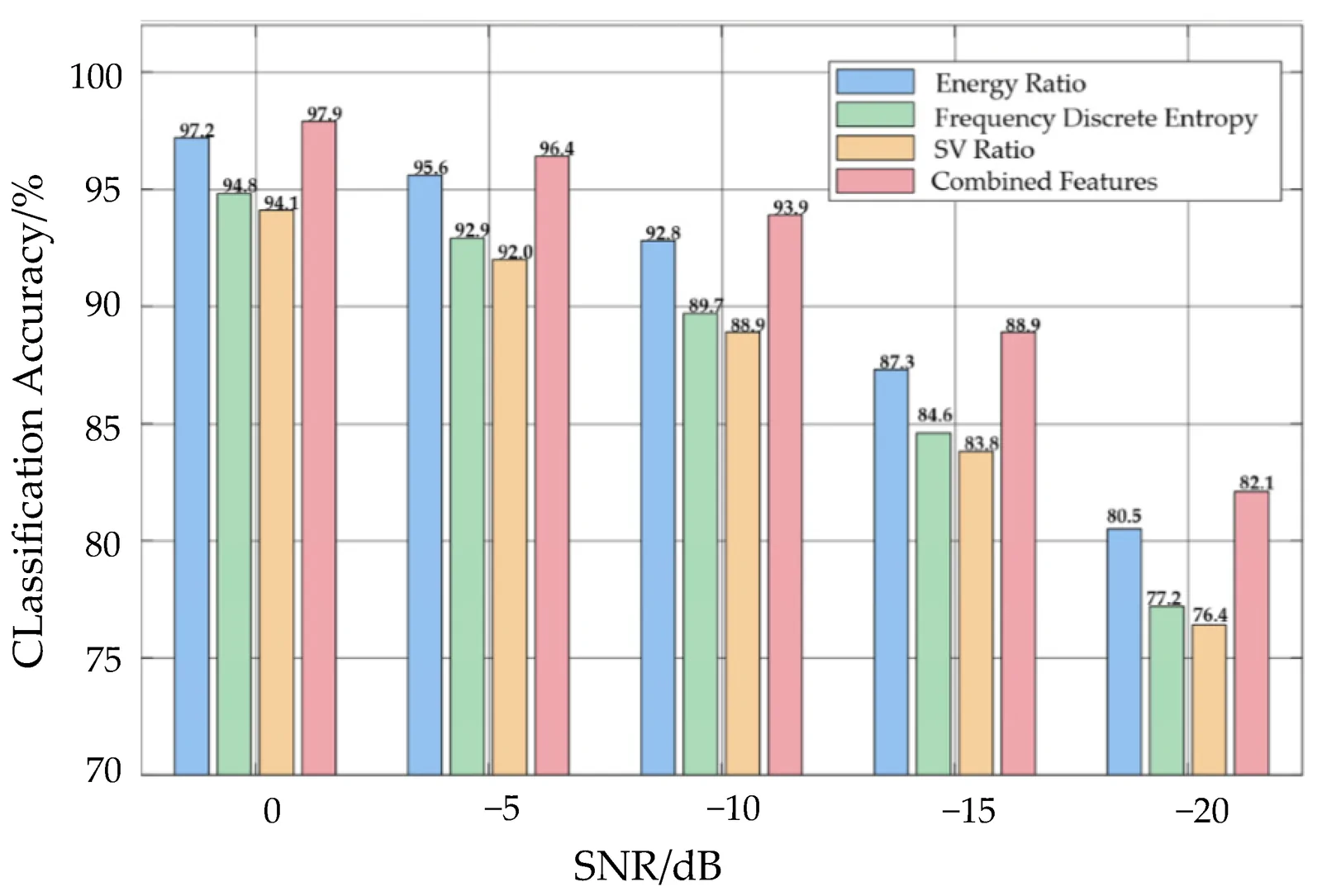
Ablation analysis of VMD-based feature combinations.

**Figure 16:**
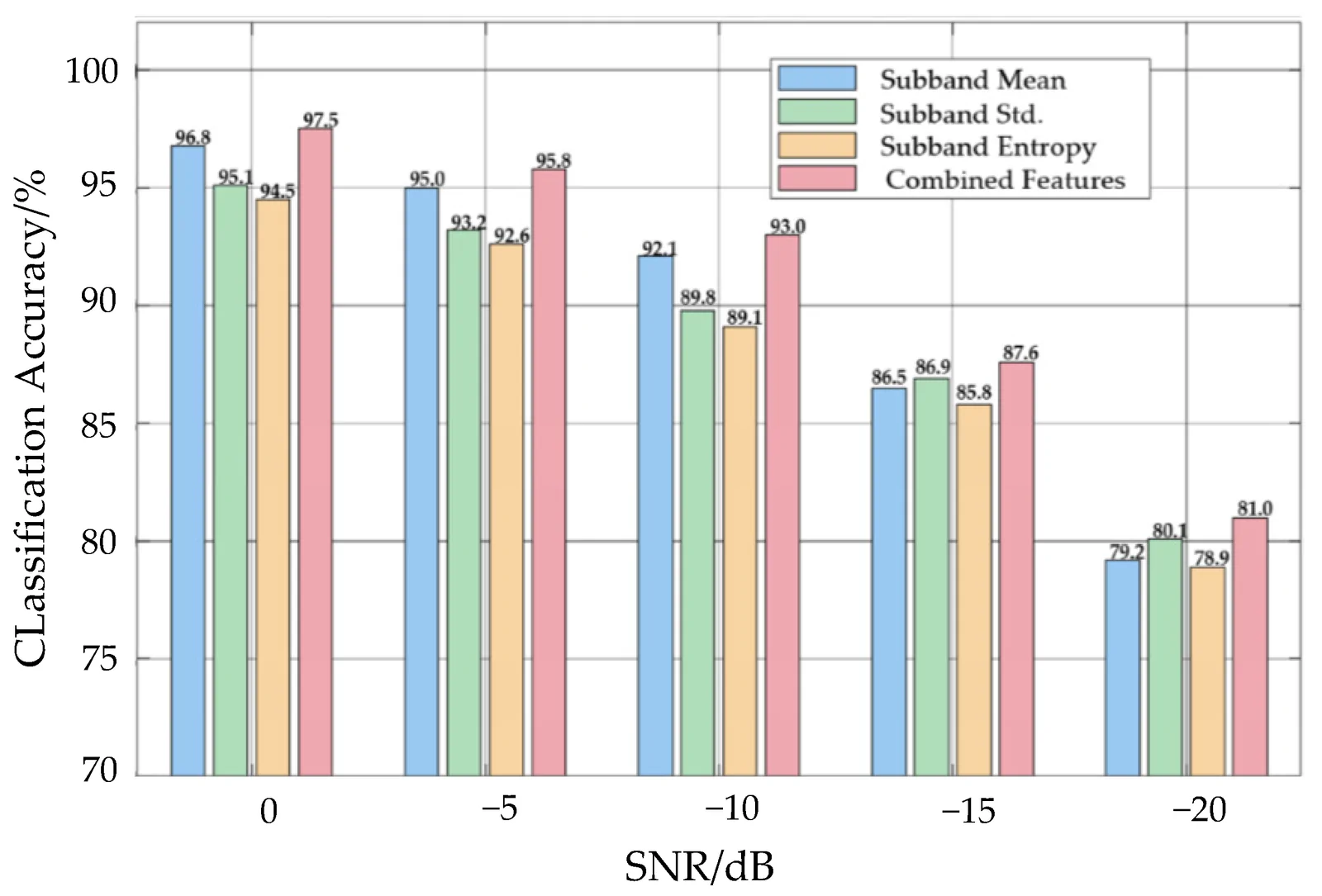
Ablation analysis of STFT-based feature combinations.

**Figure 17:**
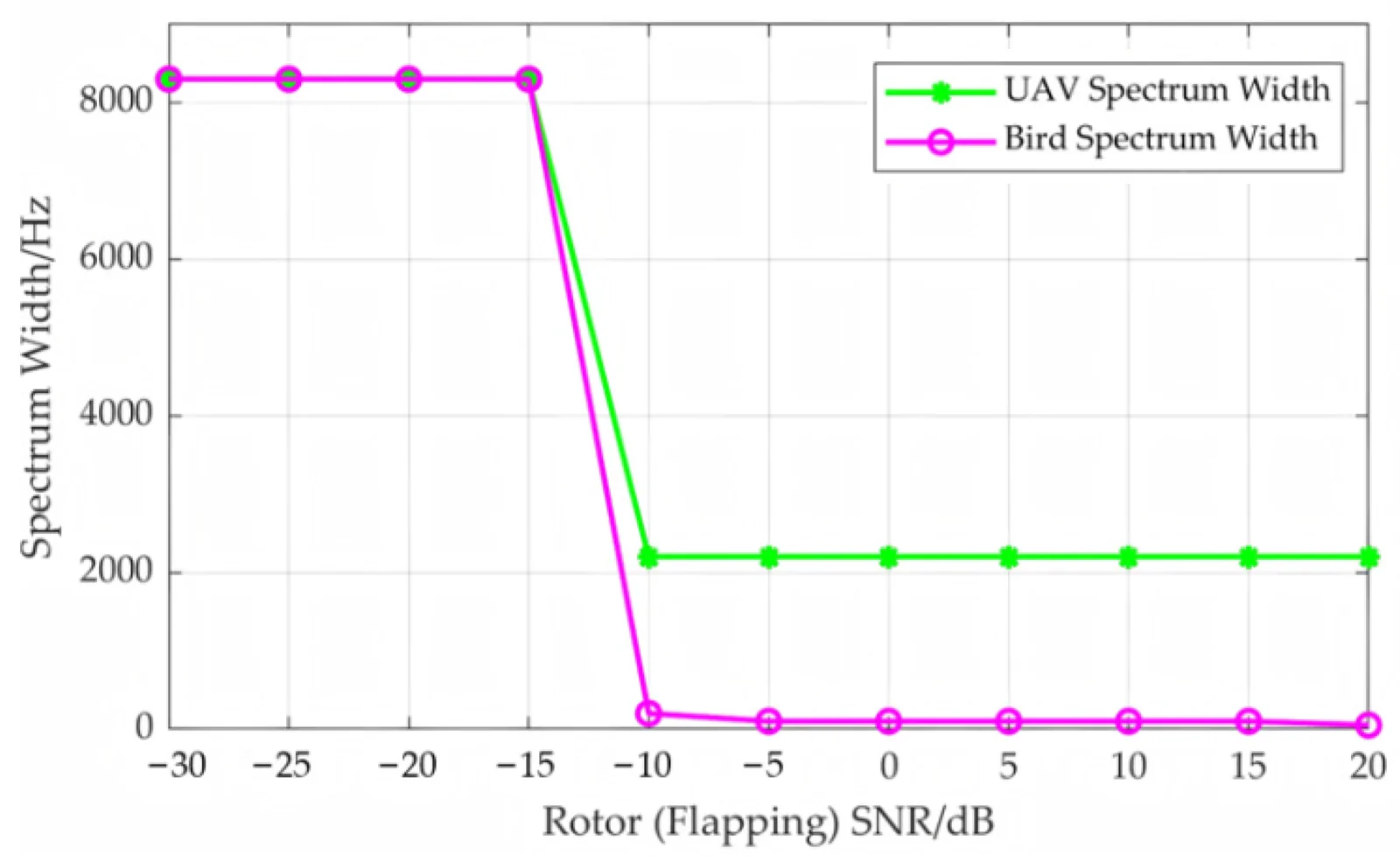
Detection results of spectral width for UAVs and birds.

**Figure 18:**
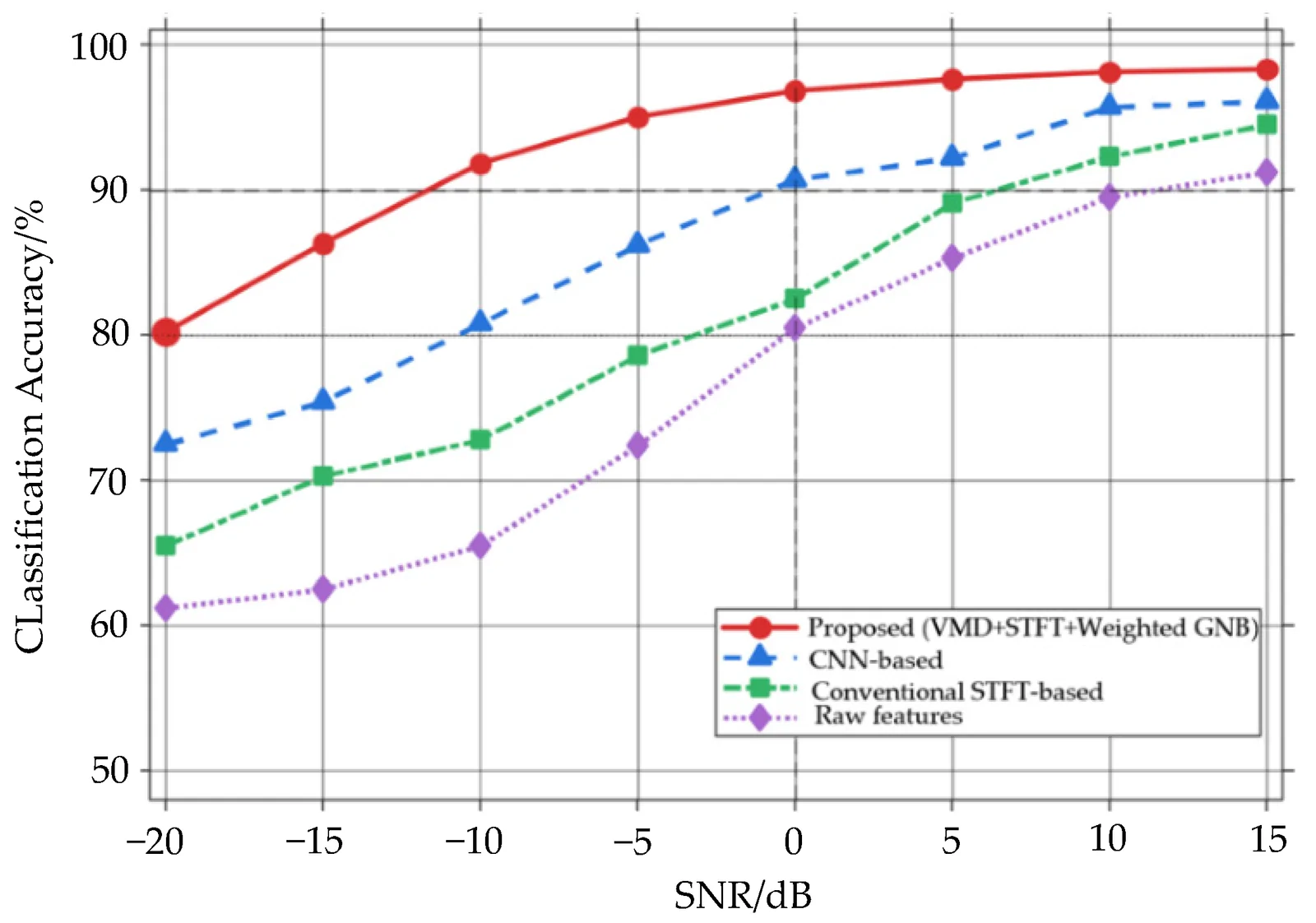
Classification Accuracy vs. SNR.

**Figure 19:**
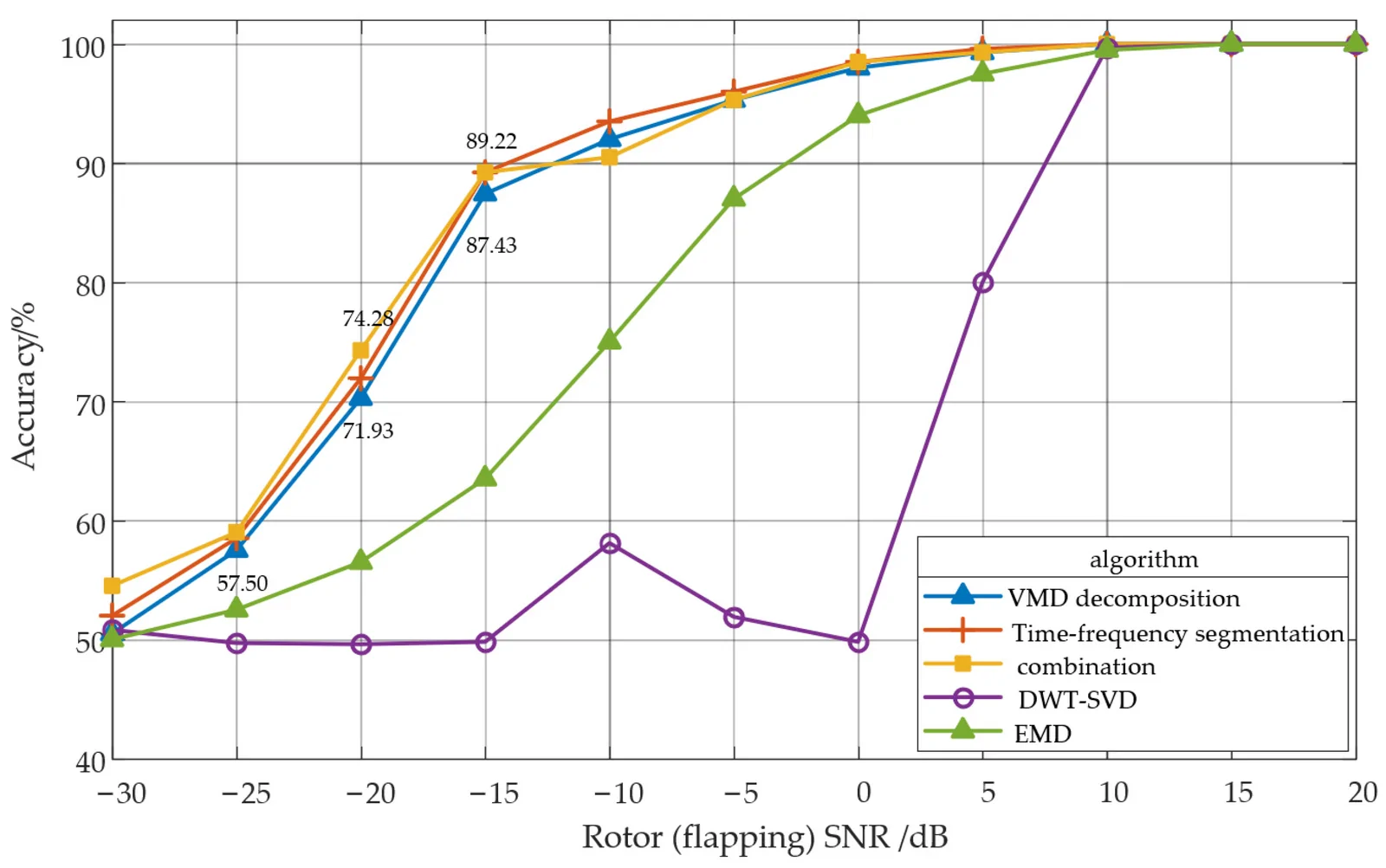
Comparison of classification accuracy of each method under different rotor (wing flap) signal-to-noise ratios.

**Figure 20:**
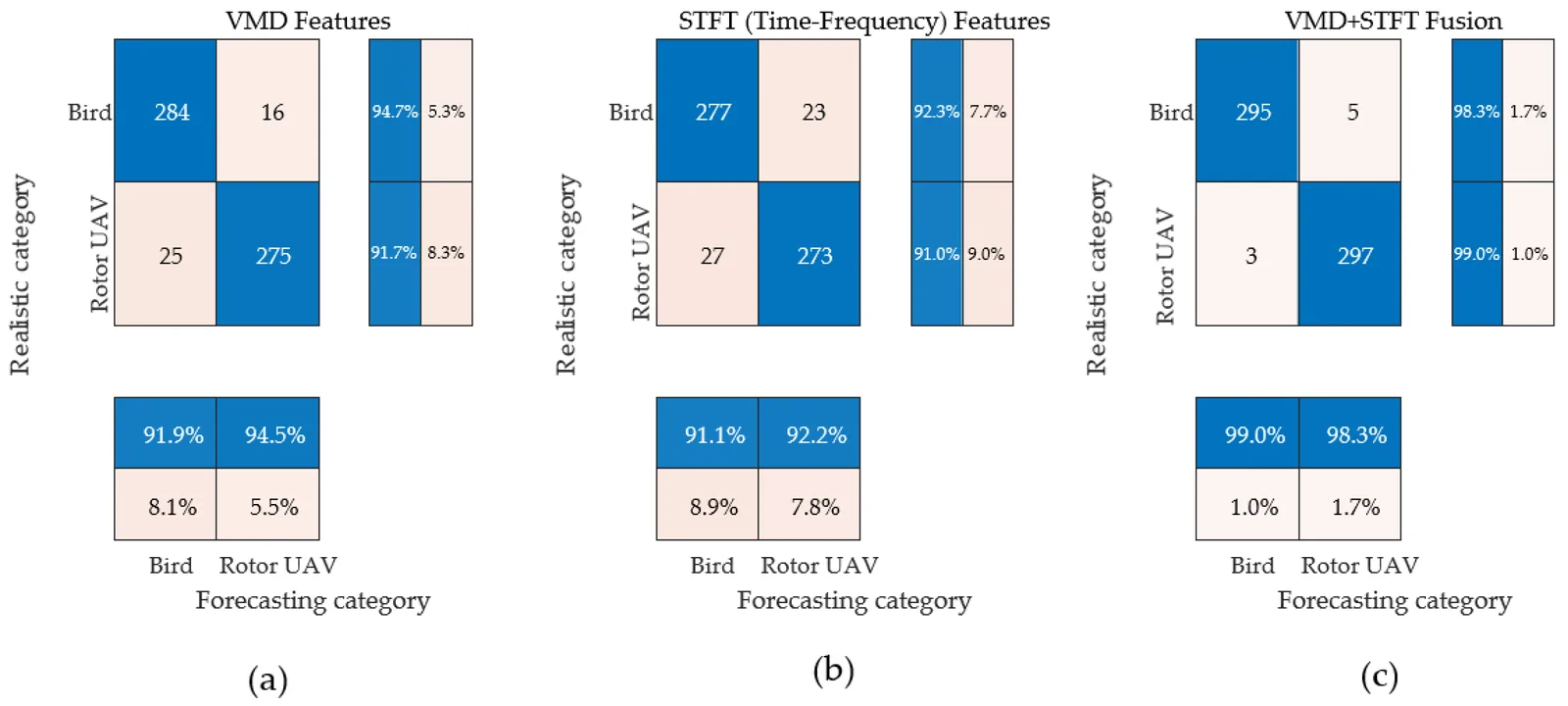
Confusion matrices of the VMD-based, STFT-based, and fused VMD–STFT feature representations at SNR = −5 dB. (**a**) VMD Features. (**b**) STFT (Time-Frequency) Features. (**c**) VMD+STFT Fusion.

**Figure 21:**
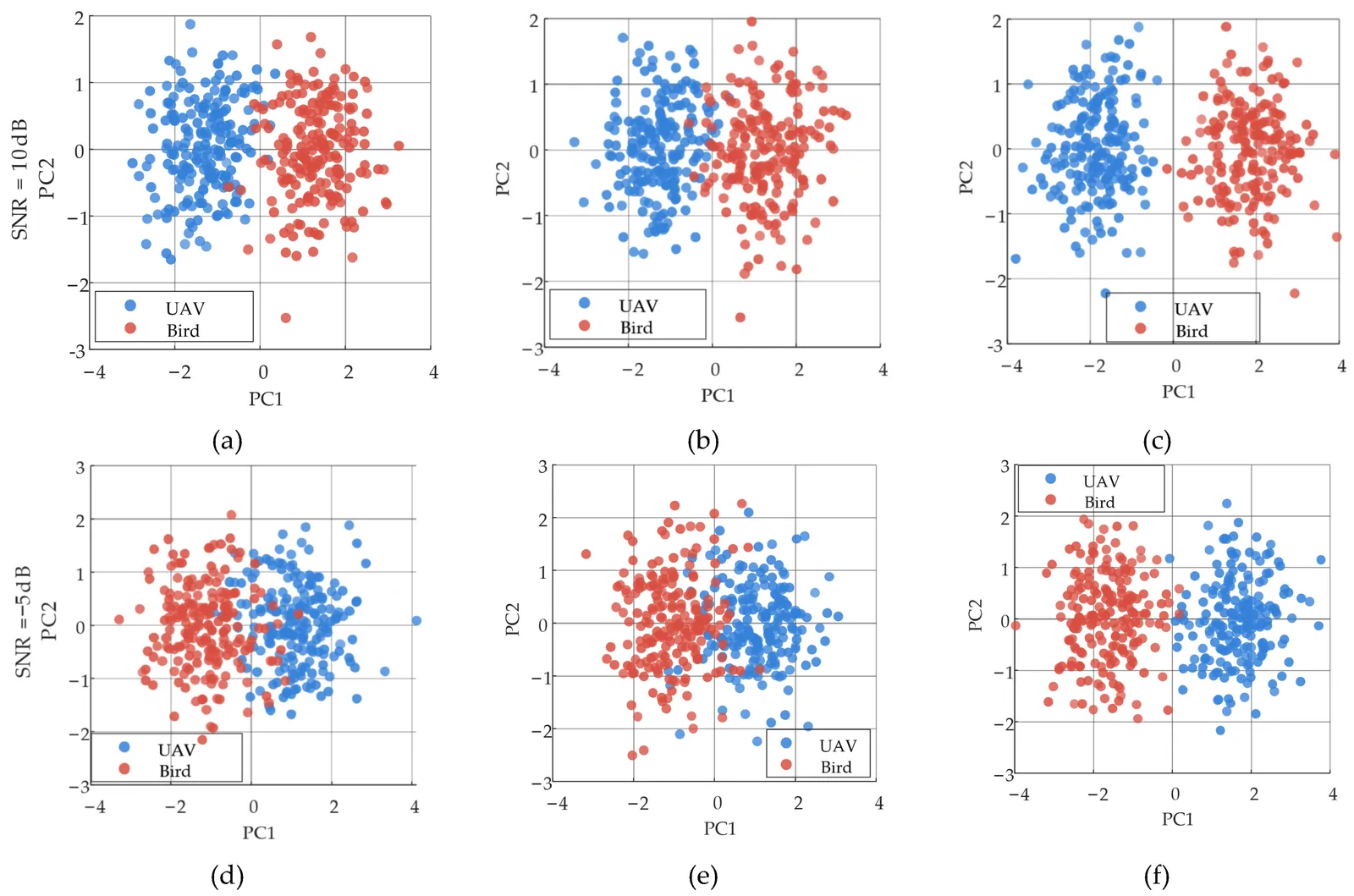
Feature-space distributions of different methods at SNR = 10 dB and SNR = −5 dB. (**a**) VMD Features (SNR = 10 dB). (**b**) STFT (Time-Frequency) Features (SNR = 10 dB). (**c**) VMD+STFT Fusion (SNR = 10 dB). (**d**) VMD Features (SNR = −5 dB). (**e**) STFT (Time-Frequency) Features (SNR = −5 dB). (**f**) VMD+STFT Fusion (SNR = −5 dB).

**Table 1:** Parameters affecting the simulation changes in quadcopter UAV and bird echo signals.

Parameter	Four-Rotor UAV	Bird
Pitch angle/°	15–55	15–55
azimuth/°	0–90	0–90
Speeds of rotors 1 and 2/(r/s)	50–110	—
Speeds of rotors 3 and 4/(r/s)	70–130	—
Wing-flapping frequency/Hz	—	5–14

**Table 2:** Radar Simulation Parameters.

Parameter	Numeric Value
Radar operating frequency/GHz	9.6
Pulse length/μs	1.2
Pulse repetition interval/μs	50
Signal bandwidth/MHz	12

**Table 3:** Parameters unchanged during simulation of the target echo signal.

Target Type	Parameter	Numeric Value
Rotary-wing UAV	Blade Length/m	0.15
	Number of blades	2
	Number of rotors	4
Bird	Radial velocity/(m/s)	8–12
	Upper Arm Length/m	0.10
	Anterior forearm length/m	0.14
	Upper Arm Swing Amplitude/°	35
	Forearm swing amplitude/°	25
	Upper arm phase lag/°	10
	Anterior forearm phase lag/°	35
	Anterior forearm rotation range/°	15
	Anterior forearm torsion lag/°	8
	Radial velocity/(m/s)	7–10

**Table 4:** Classification accuracy (%) of the VMD-based candidate feature families.

SNR/dB	Energy Ratio	Frequency Discrete Entropy	Singular Value Ratio	Joint Feature
0	96.5	94.8	94.1	97.9
−5	93.7	92.9	92.0	96.4
−10	90.8	89.7	88.9	93.9
−15	84.4	84.6	83.8	88.9
−20	78.5	77.2	76.4	82.1

**Table 5:** Classification accuracy (%) of the STFT-based candidate feature families.

SNR/dB	Subband Mean	Standard Deviation	Subband Entropy	Joint Feature
0	96.8	95.1	94.5	97.5
−5	95.0	93.2	92.6	95.8
−10	92.1	89.8	89.1	93.0
−15	86.5	86.9	85.8	87.6
−20	79.2	80.1	78.9	81.0

**Table 6:** Feature-selection and ablation results of the proposed classification framework.

Feature Combination	Feature Description	0 dB	−5 dB	−10 dB	−15 dB	−20 dB
All candidate features	Six candidate feature families before feature selection	97.8	96.2	93.1	88.5	83.2
MER only	VMD modal energy ratio feature family	96.5	93.7	90.8	84.4	78.5
Subband Mean only	STFT subband mean feature family	96.8	95.0	92.1	86.5	79.2
MER + Mean + Weighted GNB	Selected feature families and proposed final model	96.8	95.0	91.8	86.3	80.2

## Data Availability

The data that support the findings of this study are available from the corresponding author upon reasonable request.
